# When drug treatments bias genetic studies: Mediation and interaction

**DOI:** 10.1371/journal.pone.0221209

**Published:** 2019-08-28

**Authors:** Amand F. Schmidt, Hiddo J. L. Heerspink, Petra Denig, Chris Finan, Rolf H. H. Groenwold

**Affiliations:** 1 Institute of Cardiovascular Science, Faculty of Population Health, University College London, London, England, United Kingdom; 2 Department of Cardiology, Division Heart and Lungs, University Medical Center Utrecht, Utrecht, the Netherlands; 3 Department of Clinical Pharmacy and Pharmacology, University of Groningen, University Medical Center Groningen, Groningen, The Netherlands; 4 Department of Clinical Epidemiology, Leiden University Medical Center, Leiden, the Netherlands; London School of Hygiene and Tropical Medicine, UNITED KINGDOM

## Abstract

**Background:**

Increasingly, genetic analyses are conducted using information from subjects with established disease, who often receive concomitant treatment. We determined when treatment may bias genetic associations with a quantitative trait.

**Methods:**

Graph theory and simulated data were used to explore the impact of drug prescriptions on (longitudinal) genetic effect estimates. Analytic derivations of longitudinal genetic effects are presented, accounting for the following scenarios: 1) treatment allocated independently of a genetic variant, 2) treatment that mediates the genetic effect, 3) treatment that modifies the genetic effect. We additionally evaluate treatment modelling strategies on bias, the root mean squared error (RMSE), coverage, and rejection rate.

**Results:**

We show that in the absence of treatment by gene effect modification or mediation, genetic effect estimates will be unbiased. In simulated data we found that conditional models accounting for treatment, confounding, and effect modification were generally unbiased with appropriate levels of confidence interval coverage. Ignoring the longitudinal nature of treatment prescription, however (e.g. because of incomplete records in longitudinal data), biased these conditional models to a similar degree (or worse) as simply ignoring treatment.

**Conclusion:**

The mere presence of (drug) treatment affecting a GWAS phenotype is insufficient to bias genetic associations with quantitative traits. While treatment may bias associations through effect modification and mediation, this might not occur frequently enough to warrant general concern at the presence of treated subjects in GWAS. Should treatment by gene effect modification or mediation be present however, current GWAS approaches attempting to adjust for treatment insufficiently account for the multivariable and longitudinal nature of treatment trajectories and hence genetic estimates may still be biased.

## Introduction

Genome-wide association studies (GWAS) attempt to discover associations between genetic variants and a particular phenotype (an outcome)[1]. Due to an increased interest in GWAS of subjects who develop disease (see for example the ongoing Genetics of Subsequent Coronary Heart Disease consortium[2–4]) genetic analyses will more frequently be conducted using information about subjects who have received pharmacological (or other kinds of) treatments.

Because drug treatments may affect the phenotype of interest (e.g., diuretics lower blood pressure), the presence of treated subjects is typically perceived as a cause of bias in GWAS. For example, in a meta-analysis by Ehret et al.[5] blood pressure (BP) regulating drugs were used by 0.5% to 99% of the subjects enrolled in the included cohorts (median across studies: 48%). To correct for potential effects of treatment, 10 mm Hg was added to the BP measurements of treated subjects. Implicitly, such corrections assume that treatment effects do not change over time and that all subjects respond the same; both assumptions are likely incorrect.

Time-invariant corrections for treatment(s) are based on the landmark paper by Tobin[6] et al., who found that using a censored linear regression model or addition of a constant value adequately corrected for treatment-induced bias in genetic association. At the same time they warned against ignoring treatments, against excluding treated subjects, and against conditioning on received treatment. In the current manuscript we build upon Tobin’s work and generalize their work to settings were treatment status may change over time (longitudinal settings), the treatment-outcome association may be confounded, treatment may depend on the genetic variation and consider settings where treatment modifies the genetic effect.

Previous GWAS did not have access to longitudinal data on phenotypes, treatments and potential confounding factors. However, with increased linkage of genetic data to electronic healthcare records (EHR) this type of information will quickly become commonplace. To aid statistical geneticists in analysing and interpreting such enriched GWAS, we first introduce the genetic estimand (the quantity estimated) of interest. Second, we use graph theory to determine in which settings treatments may bias traditional genetic models ignoring treatment. Thirdly, using simulated data we evaluate modelling strategies of the kind previously proposed and extend these to longitudinal cohort settings (such as EHR database research). As an example, we analyse single-nucleotide polymorphisms (SNPs) on their association with glycated hemoglobin (HbA_1c_) measured over a 3-year period in a cohort of type 2 diabetes mellitus (T2DM) patients.

## Methods

### GWAS estimand

**Fig 1:**
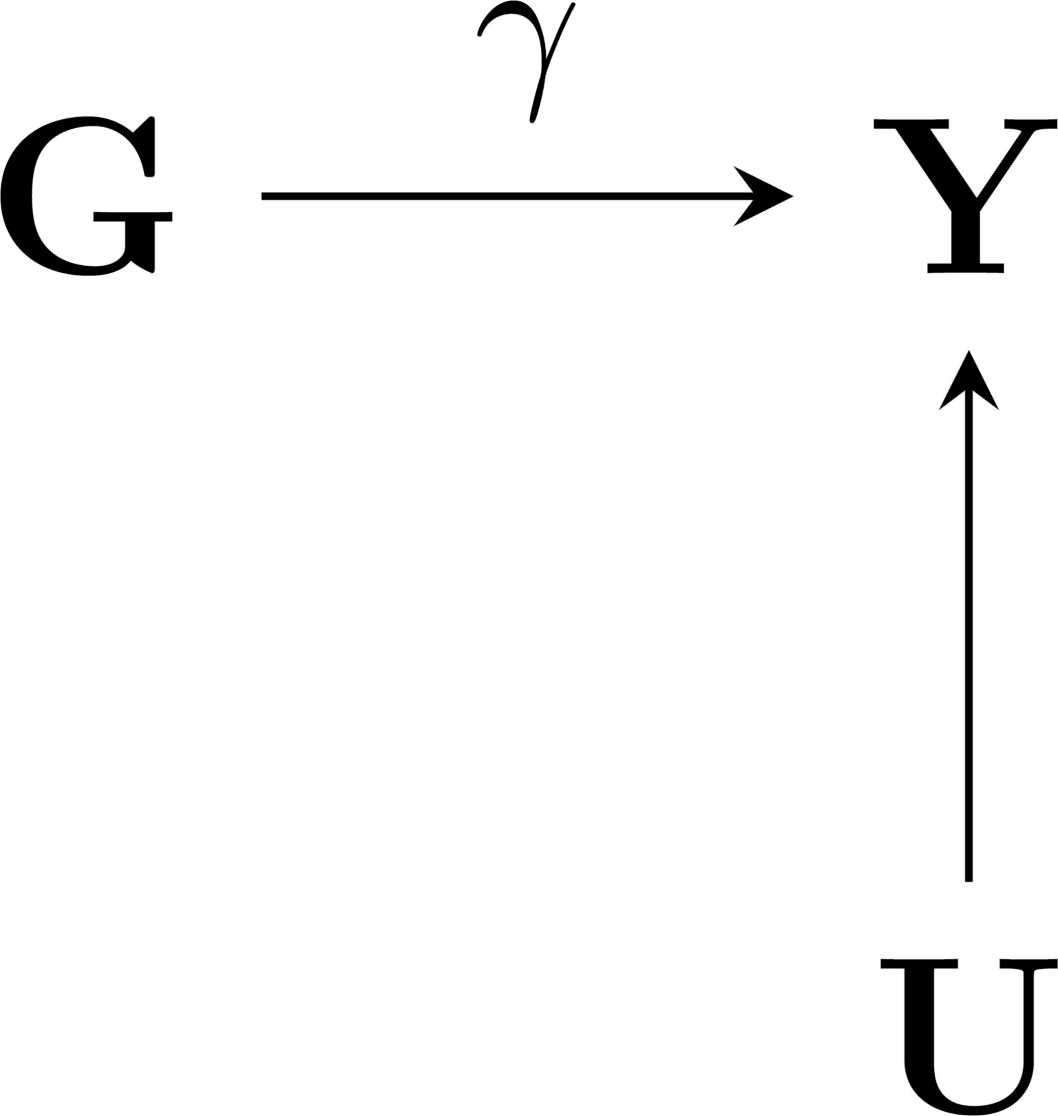
A directed acyclic cyclic graph of a genome wide association study with genetic (G) exposure, phenotype (Y), and environmental factors (U). Nb. gamma represents the magnitude of the life-time genetic association.

Fig 1 depicts a simplified directed acyclic diagram (DAG) of a possible GWAS study where a quantitative trait ***Y*** depends on a genetic exposure ***G***, and (possibly unmeasured) environmental factors ***U***. For the moment we will assume none of the enrolled subjects were treated with a drug affecting ***Y***. It is implicit in this graph that the genetic variant occurs before the phenotype, in fact a subjects’ genotype is determined at conception and often the phenotype is measured years later. In this setting *γ* represents the “life-time” effect of a genetic variant on the phenotype. Often an estimate of *γ* is obtained from *i* = 1,…,*n* samples using for example a marginal linear regression model:

$$E[Y|G]=\omega_{0}+\gamma^{*}g_{i},$$ (1)

with * to differentiate the marginal effect from a conditional effect such as presented in Fig 1. In the following we derive expressions for treatment related bias of γ* of a “traditional” marginal GWAS model set out in Eq 1.

### GWAS and treatment related bias

Obviously, between conception and the moment of phenotype measurement, many factors may influence ***Y***; one of these “environmental” factors may be a medical treatment ***D***. From Fig 1 it is clear that, should treatment simply affect ***Y*** without influencing *γ*, it is no different from any other environmental factor and both may be represented by node ***U***. In that case estimates of γ* without conditioning on ***U*** will–in expectation–equal *γ*. In other words, treatment affecting the phenotype is *insufficient* to bias the life-time variant-to-phenotype *association*.

**Fig 2:**
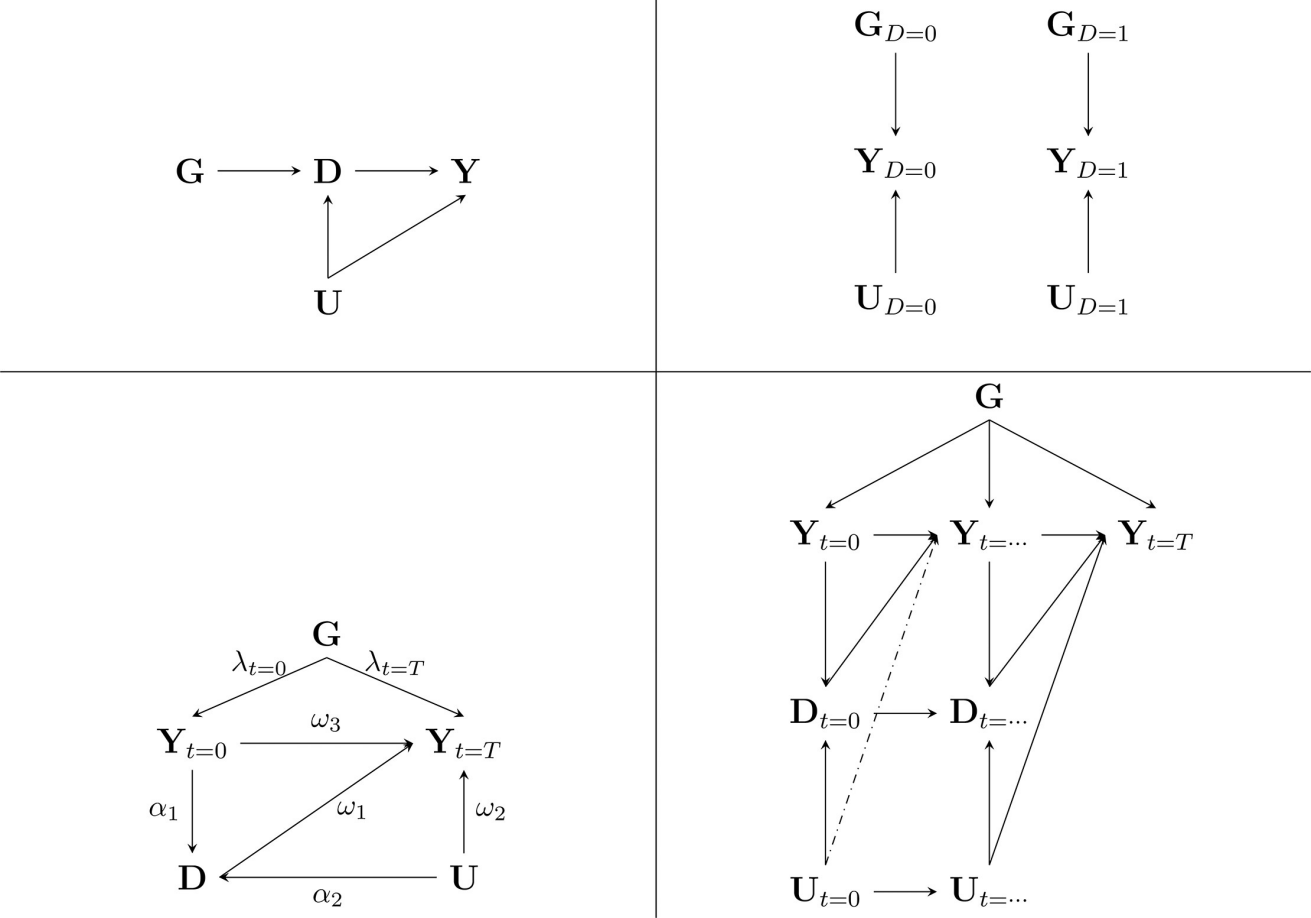
Directed acyclic graphs of treatment mediation or modification of a genetic variant-to-phenotype association. Nb. genetic (**G**) exposure, treatment (**D**), outcome phenotype (**Y**), environmental factors (**U**), and time *t*. Pathway labels are explained in the main text and appendix.

However, γ* may be biased, if treatment lies on the causal pathway between ***G*** and ***Y***; which is depicted in the top left panel of Fig 2. For example, treatment could be initiated due to the genotype increasing BP level, placing ***D*** between the genetic variant and subsequent measurements of BP. We say that treatment *mediates* the effect of ***G*** on ***Y***, and we infer from Fig 2 that ignoring treatment, such as in Eq 1, results in *E*[γ*] ≠ *y*. Similarly, but in a distinct manner, treatment may bias γ* should treatment modify the effect of ***G*** on ***Y***. For example, in the top right panel we depict two separate graphs for untreated *D* = 0 and treated *D* = 1 subjects, if we find that *γ*_*D* = 0_ ≠ *γ*_*D* = 1_ we say that there is a variant-by-treatment interaction, or equivalently, we say treatment modifies the effect of ***G*** on ***Y*** [7]. The traditional GWAS model (Eq 1) does not include such a variant-by-environment interaction, and therefore *E*[γ*] ≠ *y*. Notice that in the preceding *D* ∈ {0,1}, however, the same arguments hold should ***D*** follow a different distribution; discrete (e.g., representing multiple drugs, or counting drug prescriptions) or continuous (representing dosages).

In the previous decomposition, the phenotype and drug exposure were considered to be measured only once. Here, we define *γ*, that is the life-time effect a genetic variant has on a phenotype, when there are two phenotype measurements available, at study baseline *t* = 0, and (for example) at the end of the study *t* = *T*. As depicted in the bottom left panel of Fig 2, a drug affecting ***Y***_*t* = *T*_ might have been prescribed based on ***Y***_*t* = 0_ and ***U***.

First let us assume that a drug may have been prescribed independently of ***G***, that is *α*_1_ = 0. In this setting we let *λ*_*t*_ represent the effect of a genetic variant on the phenotype at time *t* and *ω*_3_ the effect the phenotype ***Y***_*t* = 0_ has on the subsequent phenotype measurement ***Y***_*t* = *T*_, then the *life-time* genetic effect is

$$\gamma =\lambda_{t=T}+\lambda_{t=0}\omega_{3}.$$

These terms may be estimated using, for example, separate linear regression models:

$$E[Y_{t=0}|G]=\omega_{0,t=0}+\lambda_{t=0}g_{i},$$ (2)

with intercept *ω*_0,*t* = 0_. For *t*>0:

$$E\left[Y_{t}|G,D_{-t},U_{-t},Y_{-t}\right]=\omega_{0,t}+\lambda_{t}g_{i}+\omega_{1}d_{i,-t}+\omega_{2}u_{i,-t}+\omega_{3}y_{i,-t}+\omega_{4}g_{i}d_{i,-t},$$ (3)

where the index −*t* indicates the last measurement before *t*, and *ω*_4_ allows for an interaction between treatment and the genetic variant. Here treatment is included, not to reduce bias, but merely to decrease the residual variation and hence increase power.

The above is clearly a different estimator than typically employed in GWAS (Eq 1). However, from Fig 2 and Eq 3, we infer that when there is no mediation (*α*_1_ = 0, see Fig 2) and no interaction *ω*_4_ = 0, γ* = *γ*. That is the traditional GWAS model of Eq 1 utilizing a single phenotype measurement estimates the life-time genetic effect.

In the presence of treatment related mediation (*α*_1_,*ω*_1_ ≠ 0) however, applying the traditional GWAS estimator results in an estimate which is the sum of *γ* and a “bias” term related to treatment (see the bottom left panel of Fig 2):

$$\gamma^{*}=\lambda_{t=T}+\lambda_{t=0}\omega_{3}+\alpha_{1}\omega_{1}=\gamma +\alpha_{1}\omega_{1}.$$

In addition to mediation, treatment may bias a genetic association via interaction, which occurs when *ω*_4_≠0. Assuming ***G*** does not influence the treatment decisions (i.e., an absence of mediation, *α*_1_ = 0) applying the traditional GWAS estimator results in an estimate of γ* = *λ*_*t* = *T*_ + *λ*_*t* = 0_(*ω*_3_ + *ω*_4_); that is the gene by treatment interaction biases the traditional GWAS estimate.

These decompositions show that under the strict null hypothesis of no genetic effect *λ*_*it*_ ≡ 0 for *all* subjects during the *entire* follow-up period, tests and effect estimates cannot be biased by the treatment, and the type 1 error rate will not be affected irrespective of the presence or absence of treated subjects in a GWAS.

### The genetic effect in the presence of time-varying treatment

The bottom left diagram in Fig 2 extends these scenarios to considering longitudinal settings, where both phenotype and treatment can change during follow-up; be it stopping treatment, adding treatment, or change of dosage. In such a setting the genetic association with treatment set to a reference level (e.g.,***D*** = 0) equals:

$$\gamma =\lambda_{t=T}+\sum_{t=0}^{T-1}\lambda_{t}\omega_{3t}.$$ (4)

While there are many ways to derive estimates of these terms (e.g., mixed-effect models), here we note that an estimate of the first term may be derived using the model specified in Eq 2, and *T* many estimates of second term can be derived by repeatedly applying the model of Eq 3.

### Simulation methods: treatment modelling strategies in GWAS

EHR linked to genetics will often provide sufficient data to fit the model specified in Eq 2; henceforth called the **prior to treatment** estimator. However, the model of Eq 3 often requires a fine-grained level of data which may not always be available. For example, while ***Y*** and ***D*** are frequently recorded in EHR, it is unlikely that all common causes (i.e., confounders) ***U*** of ***D*** and ***Y***, are also recorded; especially across time. Hence in typical empirical settings it may be difficult to use Eq 4 to estimate *γ* in the presence of treatment mediation or gene by treatment interaction. Therefore in simulation studies we compared a number of frequently used, or proposed, alternative treatment modelling strategies[6], requiring less data, on their ability to estimate *γ*.

The reader is referred to pages 1–5 in S1 Appendix and Table 1 for a detailed description of the modelling strategies considered. Briefly, the strategies that we will consider:

■ **Marginal model**: regressing the last measured phenotype on a genetic variant, ignoring any possibly treatment prescriptions (Eq 1); the typical model used in GWAS.
■ **Conditional model 1:** conditioning on treatment but ignoring common causes (confounders) of treatment and the phenotype; a potentially naïve attempt to model treatment in GWAS.
■ **Untreated subgroup** fitting a marginal model on a sample restricted to untreated subjects (or more generally a patient group treated with the same drug); a possible method of data preparation.
■ **Addition of a constant** adding a constant value, representing the likely treatment effect, to the phenotype of treated subjects and subsequently applying the marginal model; as suggested by Tobin et al.
■ **Censored regression** Treating the observed phenotype values of treated subjects as right-censored observations (e.g., the phenotype measurement of treated subject would have been higher if they had not been treated); as proposed by Tobin et al.
■ Performance of these less data-intensive strategies will be compared to the model presented in Eq 3 without and with a gene by drug interaction: **conditional models 2** and **3**, as well as the **prior to treatme**nt estimator (Eq 2).

**Table 1:** GWAS treatment modelling strategies considered in simulated and empirical data.

Modelling strategy	Implementation	Key assumptions
Marginal model	• Associates the phenotype to the genetic variant(s) without conditioning on other variables or accounting for longitudinal measurements (taking a single measurement for each subject).	• Assumes treatment does not modify the variant-to-phenotype association • Assumes treatment allocation is not (indirectly) influenced by the genetic variant (absence of mediation). Notably this model does **not** assume treatment does not influence the phenotype
Conditional model 1	• Associates the phenotype to the genetic variant(s), conditional on prescribed treatment(s). • Multiple period-specific models are fitted to account for the presence of longitudinal data (e.g., on the phenotype and treatment). • *γ* is estimated by summing *λ*_*t*_.	• Assumes treatment does not modify the variant to phenotype association • Assumes there are no common causes of both treatment and the phenotype (no cofounders). • Assumes previous phenotype measurements do not influence subsequent phenotype levels i.e., *ω*_3*t*_ ≡ 0
Untreated subgroup	• Stratifies the available data on an untreated group of patients and fits a marginal model to the subgroup; or more generally a group of patients with the same treatment. • Longitudinal changes are accounted for by applying this strategy *T* times, an estimate of *γ* is obtained by summing *λ*_*t*_.	• Assumes there are no common causes of both treatment and the phenotype (no cofounders). • Assumes previous phenotype measurements do not influence subsequent phenotype levels i.e., *ω*_3*t*_ ≡ 0
Addition of constant	• An (out-of-sample) estimate of the treatment effect is added to the phenotype measurement of treated subjects. • The adjusted phenotypes are modelled using period-specific marginal models. An estimate of *γ* is obtained as before.	• Only accounts for mediation not for interaction. • In longitudinal setting applying repeated period-specific marginal models assumes *ω*_3*t*_ ≡ 0.
Censored regression	• Phenotype measurements are treated as censored observations of their unobserved untreated phenotype level. • Following Tobin et al., we fit a censored regression model without accounting for covariables. The longitudinal nature of treatment is accounted for by applying period-specific models and summing *λ*_*t*_	• Assumes non-informative censoring, in the sense that (conditional on potential covariables) the phenotype distribution across treatment groups is the same. • Assumes and absence of variant by treatment interaction. • In longitudinal setting applying repeated period-specific marginal models assumes *ω*_3*t*_ ≡ 0.
Conditional model 2	• Extend the period-specific conditional 1 strategy by conditioning on common causes of treatment and the phenotype. To close any backdoor pathway also conditions on previous phenotype levels. • *γ* is estimated based on Eq 4.	• Assumes treatment does not modify the variant to phenotype association • Assumes all common causes of treatments and phenotypes were (accurately) recorded, and modelled.
Conditional model 3	• Extends conditional model 2 to allow for treatment by variant interactions.	• Assumes all common causes of treatments and phenotypes were (accurately) recorded, and modelled.
Prior to treatment estimator	• Estimates the variant to phenotype association in patient data collected before a treatment decision was made.	• Does not make assumptions on the presence or absence of mediation, interaction or common causes of treatment and phenotype. • Estimates *λ*_0_ instead of *γ*

Please see appendix methods for a more formal algebraic decomposition.

These different treatment modelling strategies were applied and evaluated based on simulated data generated following the diagrams presented in the lower part of Fig 2. Briefly, and without limiting generalizability, we made the following distributional assumptions: ***D***_*t*_ followed a Bernoulli distribution, ***G*** a trinomial distribution following the Hardy–Weinberg equilibrium, the phenotype ***Y***_*t*_ followed a first-order Markov process with the regression errors of ***Y***_*t*_ derived from a multivariate normal distribution, with ***U***_*t*_ also following a multivariate normal distribution; for a full description of the data generating model we refer the reader to pages 1–5 in S1 Appendix.

In **scenario 1** an RCT was simulated, where drug treatment was allocated at baseline *t* = 0 and phenotype measurement were available for *t* ∈ {0,1}. This RCT scenario was used to determine the impact of treatment by gene interactions, setting the interaction effect to *ω*_4_ ∈ {0.5, 1.0,…,5}, the treatment effect to *ω*_1_ = −10, and the *direct* genetic effect on ***Y*** to *λ* = 0.5. In **scenario 2** a *nonrandomized* study with *t* ∈ {0,1} was simulated by setting the ***Y***_0_ to ***D*** effect to $e^{a_{1}}=1.2$, and the confounder ***U***_0_ effect on ***D*** to $e^{a_{2}}=1.5$. The influence of the *direct* genetic effect on ***Y*** was assessed by *λ* ∈ {0.0,0.2,…,1.8} (**scenario 2.A)**. Next we set *λ* = 0.5, and the treatment effect on ***Y***_1_ was evaluated by setting *ω*_1_ ∈ {0,−4,−8,…,−36} (**scenario 2.B**). Finally, in **scenario 3** we simulated a nonrandomized study in which treatment changed over time; *t* ∈ {0,1,…,5}. In **scenario 3.A** we assumed all *t* measurements were available, in **scenario 3.B** we assumed measurements were available only at *t* ∈ {0,5}; thus (incorrectly) treating the longitudinal cohort as if it were a cohort where treatment is constant over time. We refer to pages 1–5 in S1 Appendix for a full description of the parameter values. To further explore the impact of model misspecification we repeated scenarios 1 and 2 generating ***Y*** with residuals sampled form a t-distribution with 2 degrees of freedom and under a dominant genetic model, respectively.

All simulations were repeated 10 000 times, using the statistical language R (version 3.4.0) for Unix[8], and the crch[9,10] and MASS[11] packages; results were evaluated based on bias, the root mean squared error (RMSE), coverage, and rejection rates. Recognizing that the prior to treatment estimator can never estimate *γ* (unless none of the subjects received treatment) this model was evaluated based on its ability to estimate *λ*_*t* = 0_ (the genetic effect prior to treatment initiation).

## Results

### Simulation results: Treatment modelling strategies in GWAS

**Fig 3:**
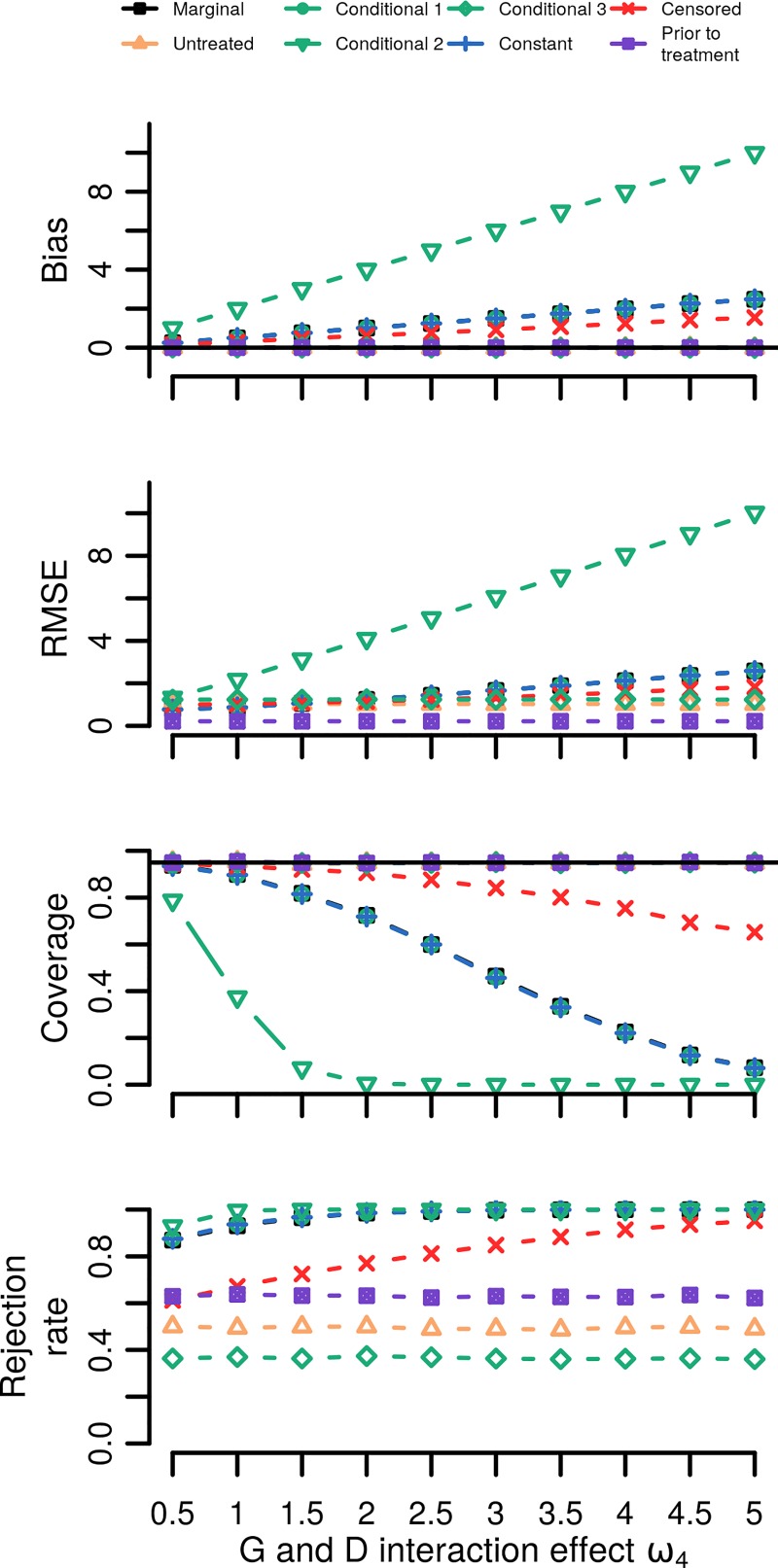
Simulation results for scenario 1 where the life-time variant-to-phenotype association was modified by treatment (a variant by treatment interaction). Nb. Estimated bias equals the estimated minus the true effect; coverage represents the proportion of times the true effect was included by the 95% confidence intervals; rejection rate the number of times the null-hypothesis of no association was rejected; the root mean squared error (RMSE) equals the square root of the squared bias + the variance of the point estimate. Simulations were repeated 10,000 times. See Table 1 for a description of the modelling strategies used.

In scenario 1, (gene by treatment interaction, no mediation) the genetic estimates were biased using the marginal model, conditional models 1 & 2, addition of a constant and censored regression strategies. On the other hand, strategies that restricted the sample to untreated subjects, that included an interaction term (conditional model 3), or that used the baseline phenotype measurement (prior to treatment) did not suffer from any bias (Fig 3). RMSE and coverage followed a similar pattern, and power (rejection rate) of the unbiased treatment modelling strategies was lowest for the conditional model 3 (36%), followed by the untreated model (50%) and finally the prior to treatment estimator (62%).

**Fig 4:**
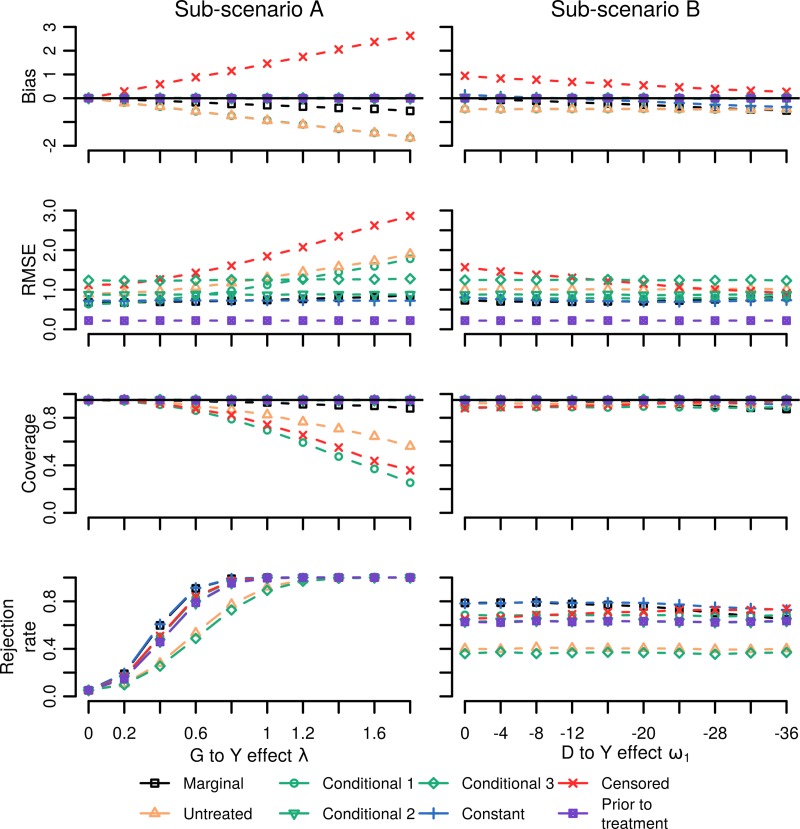
Simulation results for scenario 2 where the life-time variant-to-phenotype association was mediated by treatment. Nb. bias equals the estimated minus the true effect; coverage represents the proportion of times the true effect was included by the 95% confidence intervals; rejection rate the number of times the null-hypothesis of no association was rejected; the root mean squared error (RMSE) equals the square root of the squared bias + the variance of the point estimate. Simulations were repeated 10,000 times. In sub-scenario A the genetic effect on the phenotype was iterated, in sub-scenario B the treatment effect on phenotype was iterated. See Table 1 for a description of the modelling strategies used.

In scenario 2, treatment mediated the genetic effect on the phenotype (no variant by treatment interaction). Contrary to scenario 1, excluding treated subjects resulted in a biased genetic estimate, showing an equal amount of bias as conditional model 1, with bias of the censored regression model generally highest (Fig 4). In these settings the marginal model was biased as well, however often less than the strategies detailed above. Conditional models 2 and 3, as well as the prior to treatment model were unbiased throughout, with bias of the constant approach close to zero unless treatment effect was much larger than the assumed effect of -10 (right panel Fig 4). As before, coverage followed a similar pattern as bias, ranging from 0.95 to 0.30. In general, increasing the genetic effect also increased bias (left panel of Fig 4), while bias was relatively robust to variations in treatment effect. While conditional model 2 and 3 where both unbiased, due to addition of an interaction term (in the absence of a true interaction effect), model 3 had a slightly larger RMSE. Due to the total absence of treatment, the “prior to treatment model” had the lowest RMSE.

Sampling residuals from a t-distribution (Figs A and B in S1 Appendix) instead of the standard normal distribution increased the RMSE, as expected, which impacted coverage and rejection rate. However, relative performance of all modelling strategies remained the same. Under a dominant genetic model (with residuals sampled from a standard normal distribution), bias and RMSE increased with the genetic effect (Scenario 2 A; Figs C and D in S1 Appendix). With a modest genetic effect (0.50), the relative performance of the different modelling strategies was similar despite mis-specifying the genetic model (Scenario 1 and Scenario 2 B; Figs C and D S1 Appendix).

Scenario 3 extends the treatment mediation problem, allowing treatment allocation to vary across time. Assuming all treatment decisions were recorded and in the absence of a genetic effect on the phenotype (Table 2) coverage and type 1 error rates were close to nominal levels, and bias was absent for all strategies (as expected). In the absence of a genetic effect, the RMSE of conditional models 2 & 3 were similar in magnitude as the marginal model often employed in genetics, and smaller than that of conditional model 1, censored regression, adding a constant value, or focussing on untreated subjects. If there was a genetic effect however, all but the prior to treatment, conditional 2 & 3 modelling strategies were biased, which also markedly increased the RMSE. Given the adequate performance of the ‘constant’ strategy in scenarios 1 and 2 the observed bias under the alternative hypothesis may be surprising. This decrease in performance (of the constant and other modelling strategies) is caused by not conditioning on preceding phenotype measurement (i.e., assuming *ω*_3*t*_ ≡ 0), which results in *λ*_*t*_ quantifying the *t* specific effect *as well as* the cumulative effect of preceding periods, resulting in an overestimation of *γ*.

**Table 2:** Results from scenario 3 A evaluating different treatment modelling strategies for genetic association analyses in the presence of mediation and time-varying treatment, analysed using longitudinal data.

Modelling strategy	Genetic effect			
	*λ* ≡ 0.00		*λ* ≡ 0.50	
	Bias (RMSE)	Coverage (rejection rate)	Bias (RMSE)	Coverage (rejection rate)
Marginal	0.00 (0.24)	0.95 (0.05)	-0.70 (0.74)	0.16 (0.92)
Untreated	-0.01 (0.90)	0.95 (0.05)	2.31 (2.47)	0.27 (0.99)
Conditional 1	0.00 (0.72)	0.95 (0.05)	2.31 (2.42)	0.11 (1.00)
Conditional 2	0.00 (0.35)	0.95 (0.05)	0.00 (0.35)	0.95 (0.99)
Conditional 3	-0.01 (0.49)	0.95 (0.05)	-0.01 (0.50)	0.95 (0.85)
Constant	0.00 (0.81)	0.95 (0.05)	2.74 (2.85)	0.08 (1.00)
Censored regression	0.00 (0.99)	0.95 (0.05)	3.17 (3.32)	0.11 (1.00)
Prior to treatment	0.00 (0.22)	0.95 (0.05)	0.00 (0.22)	0.95 (0.62)

Numbers indicate bias (RMSE), coverage and (rejection rate). Scenario A assumes all changes of treatment were observed, here *λ* represents the direct genetic effect on the phenotype of interest.

In scenario 3.B (Table 3) we ignored the longitudinal nature of the data, only utilizing baseline and the end of study measurements. As expected under the null-hypothesis all modelling approached performed the same, however under the alternative, coverage decreased below 95% for all modelling strategies, save the prior to treatment estimator.

**Table 3:** Results from scenario 3 B evaluating different treatment modelling strategies for genetic association analyses in the presence of mediation and time-varying treatment, analysed ignoring the longitudinal nature of the data.

Modelling strategy	Genetic effect			
	*λ* ≡ 0.00		*λ* ≡ 0.50	
	Bias (RMSE)	Coverage (rejection rate)	Bias (RMSE)	Coverage (rejection rate)
Marginal	0.00 (0.24)	0.95 (0.05)	-0.70 (0.74)	0.17 (0.91)
Untreated	0.00 (0.34)	0.95 (0.05)	-0.70 (0.78)	0.46 (0.66)
Conditional 1	0.00 (0.24)	0.95 (0.05)	-0.70 (0.74)	0.17 (0.91)
Conditional 2	0.00 (0.24)	0.95 (0.05)	-0.70 (0.74)	0.17 (0.91)
Conditional 3	0.00 (0.34)	0.95 (0.05)	-0.70 (0.78)	0.46 (0.66)
Constant	0.00 (0.26)	0.95 (0.05)	-0.64 (0.70)	0.31 (0.90)
Censored regression	0.00 (0.34)	0.95 (0.05)	-0.58 (0.67)	0.59 (0.77)
Prior to treatment	0.00 (0.22)	0.95 (0.05)	0.00 (0.22)	0.95 (0.63)

Numbers indicate bias (RMSE), coverage and (rejection rate). Scenario B assumes treatment initiation and last biomarker are observed, with all variables (and changes) between *t* = 0 and *t* = *T* unobserved, here *λ* represents the direct genetic effect on the phenotype of interest.

### Genetic associations with HbA_1c_ in diabetic patients

As an illustrative example we analysed a sample of type 2 diabetes patients who were treated in general practices (GP) in the Northern part of the Netherlands and participated in the “Groningen Initiative to Analyze Type 2 Diabetes Treatment” (GIANTT) initiative[12]. Data were available on 280 patients who initiated treatment between 1999 and 2014, and consented to participate in a genetic sub-study (approval was sought and obtained from the institutional review board). We sought to associate 165 typed SNPs to longitudinal HbA_1c_ levels (as percentage of glycated haemoglobin). The GIANTT database contains information about prescriptions of antidiabetic treatments (ATC-codes: A10BA, A10BB, A10BF, A10BG, A10BH, A10A), as well as longitudinal data on the following *covariables* (selected based on[13]): LDL-cholesterol (LDL-C), systolic blood pressure (SBP), body mass index (BMI), serum creatinine, as well as disease histories (CVD, respiratory disease, liver disease, and cancer). After removing monomorphic SNPs, screening on Hardy & Weinberg’s equilibrium, with a frequency of less than 5%, and dropping SNPs with more than 5% missing data, 122 variants were retained for further analyses (see Table A in S1 Appendix). Variant missingness across subjects did not show excessive missingness (defined here as >5%) which would be indicative of quality control issues.

**Fig 5:**
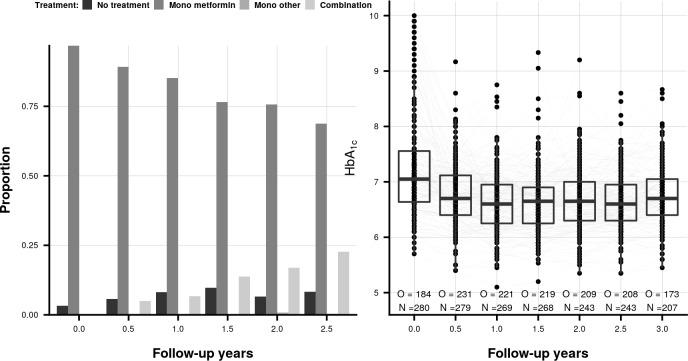
The distribution of treatment and HbA_1c_ across a 3-year follow-up period of patients enrolled in the GIANTT cohort. Nb. follow-up year 0 indicates the baseline period; the grey lines in the right panel depict individual HbA_1c_ trajectories; O and N represents the number of observed measurements compared to the number of available subjects.

We first explored treatment and biomarker trajectories across time (Fig 5, and Appendix), with longitudinal changes captured by 6-month windows. Most patients started with metformin, slowly moving to other glucose lowering treatments, or combination therapies (including insulin). HbA_1c_ markedly decreased after baseline and on average stayed at a constant level below 7% (the typical therapeutic target level). As indicated by the grey lines in Fig 5 within-subject variation was modest (presumably due to GP monitoring), more pronounced within-subject variation was observed for LDL-C, SBP, and creatinine (Figs E and F in S1 Appendix).

**Fig 6:**
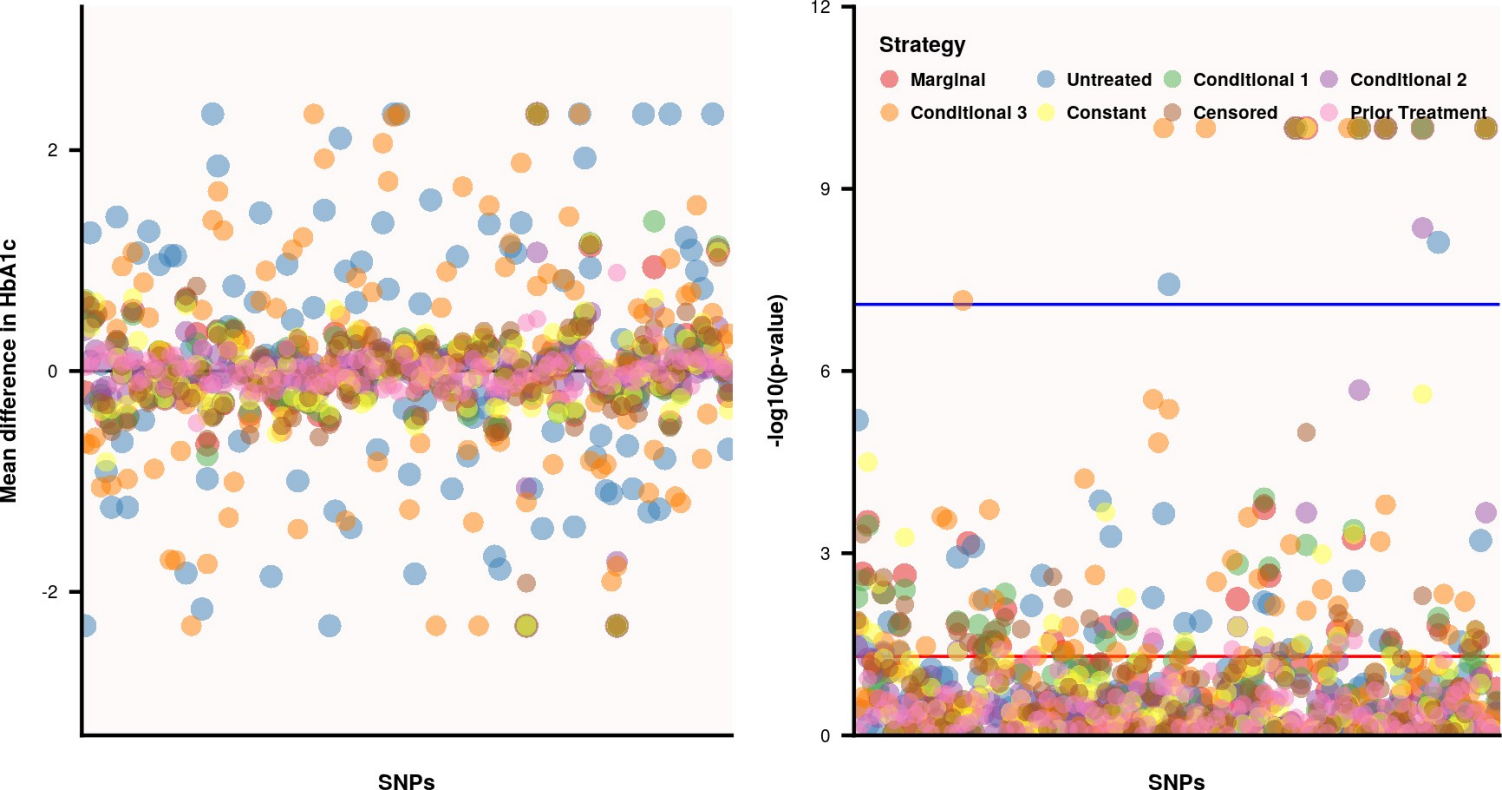
The mean difference and p-values of 122 SNPs associated to longitudinal HbA_1c_ measurement utilizing different modelling strategies to account for longitudinal changes in treatment, and covariables. Nb. The horizontal blue and red lines indicated a -log_10_ p-value of 8^−10^ and 0.05, respectively. Based on[15] the Constant estimator was implemented by adding 1 to any HbA_1c_ measurement related to the subjects receiving glucose lowering medication. Extreme values were truncated. See Table 1 for a description of the modelling strategies used.

Depending on the biomarker, the amount of missing observations could be substantial (Fig 6 and Figs E through G in S1 Appendix). We explored whether HbA_1c_ missingness was related to the available genetic variation, not observing systematic dependencies (Fig H in S1 Appendix). Associating missingness to longitudinal biomarker values and treatment, revealed a trend between observed HbA_1c_ and allocated treatment at baseline and the first year of follow-up (Table B in S1 Appendix). While these preliminary analyses did not indicate an alarming missing data problem, we nevertheless decided to impute missing values using the mice package[14]. Clustering within patient was accounted for using a random-intercept, and follow-up periods were treated as random effects. Before implementing the imputation algorithm, 76 subjects without *any* HbA_1c_, LDL-C, BMI, SBP, or creatinine measurement during the entire 3-year period were excluded, resulting in a sample of 204 subjects.

In the subsequent genetic analyses of HbA_1c_, outcome HbA_1c_ values we used from *t*+1 (preventing mixing cause and effect, for example when modelling treatment). To provide a clearer focus on the underlying modelling strategies, and limit the number of sparse cells, we chose to simplify the analyses by grouping treatments (after imputation) into “no drug treatment”, “metformin monotherapy” and “other drug therapies”. Furthermore, we did not consider drug dose.

The average genetic effect on HbA_1c_ was close to zero for all variants, irrespective of the treatment modelling strategy (right panel of Fig 6 and Table C in S1 Appendix). In untreated subjects the average mean difference was slightly higher (0.12), however so was its standard deviation: 1.20 for the untreated strategy compared to at most 0.65 for most other models. In Table 4 we focus on the 12 variants that passed the genome-wide threshold; irrespective of the estimator function. From the simulations we know that type 1 error will not be inflated under the strict null-hypothesis, so these variants are likely true positive results (provided future replication). However, rs7957197 was only detected when focussing on untreated subjects, in the current analysis this strategy often failed (due to low frequency of untreated subjects) and hence one may question these results. Barring this variant, we find that point estimates of conditional model 2 (which was often unbiased in the simulations) was typically similar to estimates from the marginal model indicating an absence of treatment mediation. Under conditional model 3 we find four variants (rS11920090, rs243088, rs4253762, rs6959643) that did not reach significance otherwise, indicating the possible presence of treatment-by-variant interaction. In general, we find some indication for treatment modification (which required independent replication, especially considering the skewed treatment distribution), with the overall agreement between modelling strategies suggesting an absence of treatment mediation of the genetic effect on longitudinal HbA_1c_ measurements.

**Table 4:** The mean difference and -log_10_(p-value) of the genetic variants with longitudinal bA_1c_ that passed the genome wide significance threshold of 8×10^−8^ under any of the proposed treatment modelling strategies.

Variant	Minor Allele	Frequency	Marginal	Untreated	Conditional 1	Conditional 2	Conditional 3	Constant	Censored	Prior Treatment
rs11920090	A	0.14	-0.17	0.20	-0.13	-0.02	-2.57	-0.20	-0.25	0.06
			(3.9x10^-1^)	(7.7x10^-1^)	(5x10^-1^)	(8.9x10^-1^)	(6.9x10^-8^)	(3.4x10^-1^)	(2.5x10^-1^)	(6.5x10^-1^)
rs243088	T	0.50	0.03	2.33	0.02	0.12	2.30	0.00	0.03	-0.13
			(8.3x10^-1^)	(2.2x10^-4^)	(9x10^-1^)	(2.3x10^-1^)	(7.5x10^-13^)	(9.8x10^-1^)	(8.6x10^-1^)	(1.6x10^-1^)
rs2447090	G	0.32	0.06	2.92	0.01	0.11	2.44	0.17	0.05	-0.19
			(7.1x10^-1^)	(3.8x10^-8^)	(9.6x10^-1^)	(4.3x10^-1^)	(4.3x10^-6^)	(3.1x10^-1^)	(7.7x10^-1^)	(9.8x10^-2^)
rs4253762	G	0.09	-0.01	-	-0.02	0.11	-2.50	0.03	0.08	-0.06
			(9.5x10^-1^)	-	(8.8x10^-1^)	(3x10^-1^)	(2.6x10^-12^)	(8.6x10^-1^)	(5.9x10^-1^)	(6.9x10^-1^)
rs6008976	A	0.49	-2.35	-	-2.44	-1.06	-1.19	-2.63	-1.92	0.44
			(1.2x10^-75^)	-	(1.3x10^-74^)	(1.6x10^-32^)	(4x10^-2^)	(1.6x10^-72^)	(8.8x10^-44^)	(5.1x10^-1^)
rs6519979	C	0.50	2.94	-	2.86	1.07	0.77	2.66	3.37	0.47
			(1.6x10^-166^)	-	(7.3x10^-4^)	(2.2x10^-4^)	(8.8x10^-3^)	(1.1x10^-141^)	(1x10^-5^)	(6.2x10^-1^)
rs6959643	T	0.17	0.39	-	0.40	0.18	2.37	0.39	0.40	0.12
			(2.2x10^-1^)	-	(2.1x10^-1^)	(3.8x10^-1^)	(4.3x10^-30^)	(3x10^-1^)	(3.2x10^-1^)	(3.8x10^-1^)
rs6963810	G	0.42	1.14	0.93	1.16	0.53	-0.82	1.16	1.14	0.13
			(7.1x10^-14^)	(1.6x10^-1^)	(8.6x10^-15^)	(2.1x10^-6^)	(2.4x10^-1^)	(3.4x10^-11^)	(2.8x10^-11^)	(4.7x10^-1^)
rs73886756	A	0.50	-4.66	-	-4.74	-1.73	-1.77	-4.93	-4.23	0.89
			(2.8x10^-208^)	-	(3.1x10^-209^)	(1.8x10^-69^)	(1.6x10^-4^)	(2x10^-195^)	(1.2x10^-141^)	(3.4x10^-1^)
rs784888	C	0.49	0.94	-	1.36	0.57	-0.72	0.66	0.39	0.33
			(1.8x10^-13^)	-	(2.1x10^-26^)	(4.4x10^-9^)	(1x10^-1^)	(2.4x10^-6^)	(5x10^-3^)	(6.2x10^-1^)
rs7957197	A	0.17	-0.47	2.59	-0.48	-0.11	1.02	-0.37	-0.51	-0.09
			(1.5x10^-2^)	(7.6x10^-9^)	(1.2x10^-2^)	(4.1x10^-1^)	(7.3x10^-2^)	(6.9x10^-2^)	(2x10^-2^)	(4.7x10^-1^)
rs9470794	C	0.08	1.10	-	1.13	0.40	0.13	1.08	1.03	0.09
			(7.5x10^-15^)	-	(1x10^-15^)	(2.2x10^-4^)	(7.5x10^-1^)	(1.9x10^-12^)	(1.5x10^-11^)	(6x10^-1^)

n.b. the “untreated” modelling strategy failed for 25 out of 122 genetic variants, with none of the other strategies failing.

## Discussion

In this paper we showed that results from genetic quantitative trait analyses performed on (partially) treated subjects may be biased when treatment status (directly or indirectly) mediates the genetic association, or when this association is modified by treatment. In such settings, ignoring treatment may bias estimators of genetic effects and yield sub-nominal coverage. Simply conditioning on treatment is unlikely to remove bias (and may actually increase bias) unless information is available on common causes (confounders) of treatment and the study phenotype. In the absence of treatment related mediation or variant-by-treatment interaction, the possibility that a drug might affect a phenotype is insufficient to bias GWAS results of the phenotype association. This contradicts a commonly held belief, that the mere presence of treatment affecting the phenotype of interest invalidates GWAS. Similarly, under the null-hypothesis of no genetic effect, GWAS are unbiased by treatment. In an empirical analysis relating genetic variations to longitudinal measurements of HbA_1c_ we observed limited evidence for treatment induced bias of genetic associations.

Previously, Tobin[6] et al. found that censored linear regression or addition of a fixed value to the phenotype measurement of treated subjects, adequately corrected for treatment-induced bias in genetic analyses. At the same time, they warned against ignoring treatment all together, excluding treated subjects, or conditioning on received treatment. The authors come to these recommendations using a cyclic (feedback) data generating model, where treatment is initiated based on a biomarker value, while at the same time decreasing the biomarker on which treatment was initiated (Fig I in S1 Appendix). We have adapted Tobin’s scenario to explicitly differentiate between the phenotype levels causing prescription and the phenotype level after treatment initiation, with further extensions considering the longitudinal settings typical of most patient histories. At the same time, we introduced issues that are likely encountered in empirical settings, such as confounding of the treatment-outcome association, mediation of the genetic effect by treatment, and gene-by-treatment interaction. Based on our analyses we concur with Tobin that ignoring treatment allocation may bias genetic analyses when treatment is (indirectly) affected by a genetic variant (or modifies its association). Similarly, we agree that in such setting simply conditioning on treatment is insufficient (and may worsen bias) to account for treatment related bias. However, as Tobin et al. eludes to as well, similar remarks may be made about applying an unconditional censored regression model in the presence of informative censoring. Generally, strong parametric decisions are necessary when modelling treatment in GWAS, for example, which potential confounders to consider, how to model time, what is the influence of previous phenotypes measurements, and so on. Furthermore, depending on the applied setting, certain combinations of the proposed strategies may perform better. For example, the censored regression model may be extended by including covariables associated with the phenotype (similar to conditional model 2) increasing the likelihood of the non-informative censoring assumption.

While we focussed on the biasing potential of drug treatments in GWAS, this is only a specific example of the more general possibility of an environmental (non-genetic) variable biasing GWAS via mediation or interaction. We belief the data generating model as presented here closely approximates empirical settings where treatment(s), as well as determinants of treatments and outcome, may change over time. We did not however, attempt to mimic any specific disease-treatment combination. As such, we do not claim the simulation parameters resemble a specific empirical setting, nor do we claim the absolute performance of the different strategies necessarily reflect (the numerous possible) empirical settings. We do however expect the *relative* performance of the evaluated strategies to be similar in empirical data. For example, (unconditional) models making strong parametric assumptions on the absence of mediation, interaction, or confounding, will often perform worse than models allowing for such effects (at the potential cost of an increased variance, e.g., comparing conditional model 2 versus 3).

We have purposely provided a detailed description of the data preparation steps of our empirical example, highlighting problems often encountered in longitudinal data. Despite having access to electronic healthcare records (EHR) from a closely monitored group of patients (such as T2DM subjects) several biomarkers measurements were missing over time. As far as these missing observations reflect measurements truly unavailable to prescribing healthcare professionals, missingness might not bias genetic-estimates (e.g., when the missing data are “missing completely at random”; MCAR). Despite the possible MCAR mechanism, we decided to impute missing observations to retain the modest sample size available. As an illustration, we focussed on a single imputed dataset, but fully recommend multiple imputation, or other methods that correctly account for imputation related variance deflation. Furthermore, due to sample size constraints, we simplified treatment measurements, grouping distinct therapies and ignoring difference in dose. In larger cohorts, such as the UK biobank, the proposed parametric models can be readily extended to account for additional treatments and dosage. For example, changes in dosage can be accommodated for by replacing treatment with a continuous variable indicating the prescribed daily drug dose. We emphasize that researchers should not only consider the type of potential confounders, but also the timing of its measurement. For example, we decided to only include biomarkers from the previous 6 months (following a first order Markov process) as confounders, however, and depending on the disease and treatment, information from additional periods may be relevant as well. Similarly, for certain pathologies previous phenotype levels (or more generally states) may not be expected to independently influence the subsequent level or state.

As mentioned throughout this manuscript, depending on the phenotype and treatment of interest, it may be impossible to collect sufficient data to use conditional models 2, and 3; which performed best in our simulated scenarios. Even in linked electronic healthcare databases with national coverage, such as found in Estonia or Denmark, information on causes of treatment initiation (or changes) are likely imperfectly collected or measured. Nevertheless, we feel these proposed modelling strategies are relevant because they delineated that more simplistic, less data intense, approaches currently applied will often perform worse. Despite the likely absence of sufficient data, applying conditional models 2, or 3, may still provide useful information on the likelihood of bias due to mediation or interaction, for example as implemented in our empirical example on genetic association with HbA_1c_. Should results suggest that treatment interaction or mediation are relevant for the genetic association of interest, researchers may consider repeating gene/variant-specific analyses using data from RCTs, where common causes of treatment and the phenotype are absent by design. Depending on the study aim, a reasonable alternative could be to focus on phenotypes measured prior to treatment initiation. This advice should however not be confused with recommending stratification on untreated subjects, which would induce confounding bias. Instead, a prior to treatment analyses entails finding a time (period) were most/all subjects did not receive treatment (for example, before diagnosis).

In conclusion, we showed that treatment may bias genetic associations with quantitative traits if the genetic effect is mediated by treatment or in the presence of a gene-by-treatment interaction. To appropriately account for such bias, genetic studies should more frequently be conducted within electronic healthcare databases, providing greater detail on the longitudinal nature of treatment and phenotype. In the absence of treatment related mediation or interaction, the genetic association obtained from a marginal linear regression model (traditionally used in genetic analyses) is expected to perform adequately.

## Supporting information

S1 Appendix Supplemental methods and results. (PDF)
