# Supplementary material for: When drug treatments bias genetic studies: Mediation and interaction
Source: PLoS One. 2019 Aug 28;14(8):e0221209. doi: 10.1371/journal.pone.0221209 (PMC6713387; doi:10.1371/journal.pone.0221209)
Supplement: S1 Appendix — (PDF) [file pone.0221209.s001.pdf]

Supplemental Material

1 Treatment modelling strategies in GWAS

As shown in Figure 2 in the main text of the manuscript, a longitudinal estimator of the variant ($\mathbf{G}$) life-time effect γ on a phenotype ($\boldsymbol{Y}$ i.e., an outcomes) can be defined as:

$$\gamma = \lambda_{t=T} + \sum_{t}^{T-1} \lambda_t \omega_{3t}. \tag{A1}$$

Here $\lambda_{t=T}$ represents the direct effect of the gene on the phenotype towards the end of follow-up, λ_t the effect of the gene at time t, and ω_{3t} the effect of $\boldsymbol{Y}_{-t}$ on $\boldsymbol{Y}_t$. Depending on the data generating model (see Figure 2 bottom right panel, and below) these terms can be estimated, for example, using the (linear regression) models:

$$\mathbb{E}\left[\boldsymbol{Y}_{t=0}|\boldsymbol{G}\right] = \omega_{0,t=0} + \lambda_{t=0} g_i, \tag{A2}$$

for $t > 0$:

$$\mathbb{E}\left[\boldsymbol{Y}_t|\boldsymbol{G}, \boldsymbol{D}_{-t}, \boldsymbol{U}_{-t}, \boldsymbol{Y}_{-t}\right] = \omega_{0t} + \lambda_t g_i + \omega_{1t} d_{i,-t} + \omega_{2t} u_{i,-t} + \omega_3 y_{i,-t} + \omega_{4t} g_i d_{i,-t}, \tag{A3}$$

with phenotype $\boldsymbol{Y}$, genetic variant $\boldsymbol{G}$, drug treatment $\boldsymbol{D}$, and environmental factors $\boldsymbol{U}$. Notice that we assume $\boldsymbol{G}$ to stay constant over time and hence omit any t index. The model defined in equation A3 allows for mediation as well as interaction. Hence model A1 can provide estimates of γ conditional on $D = 0$; for example, a reference treatment, or an untreated group.

Depending on the available data, for example genetically linked electronic healthcare records (EHR), an estimate of the first term of equation A1 (the genetic effect *prior to treatment*) may be relatively easily obtained; e.g., using the model of equation A2.

Unbiased estimates of λ_t and ω_{3t}, however require information on common causes of both $\boldsymbol{D}$ and $\boldsymbol{Y}$ which may not be recorded perfectly. In the following we therefore consider less data-intensive *treatment modelling strategies*, some of which have been previously employed in GWAS, in an attempt to account for potentially treatment-related bias of the genetic estimate(s). These strategies, often implicitly attempt to estimate γ, and are evaluated in the main text.

Marginal model

$$\mathbb{E}\left[\boldsymbol{Y}_T|\boldsymbol{G}\right] = \omega_{0T} + \lambda_T g_i.$$

This marginal model is essential equivalent to ignoring treatment entirely. In the absence of treatment related mediation or modification of the variant-to-phenotype effect, $\hat{\lambda}_T$ would provide and unbiased estimate of γ. Here we simply take the last available measurement T to represent the genetic life-time effect, in most empirical settings however, due to missing observations across time, $\boldsymbol{Y}_T$ is typically a collection of the last *available* measurement across time.

Conditional model 1

$$\mathbb{E}\left[\boldsymbol{Y}_t|\boldsymbol{G}, \boldsymbol{D}_{-t}\right] = \omega_{0t} + \lambda_t g_i + \omega_{1t} d_{i,-t}.$$

This model attempts to correct for any potential mediation of the variant-to-phenotype association by conditioning on treatment. In the presence of longitudinal changes, λ can be estimated across time resulting in multiple estimates of λ_t. The sum $\sum_{t=1}^{T} \lambda_t$ equals γ if one is willing to assume $\omega_{3t} \equiv 0$, as well as the absence of gene by treatment interactions, and an absence of common causes of both $\boldsymbol{D}$ and $\boldsymbol{Y}$. Notice that would these specific assumptions hold, especially $\omega_{3t} \equiv 0$, the term $\lambda_t = T$ (equation A1) is no longer necessary because previous values of Y will not affect subsequent observations.

Untreated subgroup

$$\mathbb{E}\left[\boldsymbol{Y}_t|\boldsymbol{G}, \boldsymbol{D}_{t=0}\right] = \omega_{0t} + \lambda_t g_i,$$

by stratifying on untreated subjects (or equivalently on a certain constant value of $\boldsymbol{D}$) this model accounts for any mediation as well as interaction. This model does implicitly assume an absence of common causes of $\boldsymbol{D}$ and $\boldsymbol{Y}$, and that $\omega_{3t} \equiv 0$.

Addition of a constant

As suggested by Tobin et al. [1], to account for the treatment effect on a phenotype one could simply add the expected treatment effect (a constant) to the observed phenotype measurements of treated subjects. Subsequently a *marginal model* could be fit to these data using the adjusted phenotype measurement, closest to the moment of treatment, as the dependent variable. To generalize this to time-varying treatment settings we simply repeat this process T times, where $\gamma = \sum \lambda_t$. In the simulations (see below) the constant was set to 10 representing the simulated treatment effect (unless this was purposely iterated).

Censored regression

Related to the previous modelling strategy Tobin et al., suggested one could treat phenotype measurements of treated subjects as right-censored observations and fit a marginal model using a censored linear regression. As before, an estimate of γ could be obtained based on the sum of estimates of λ_t.

Further models

These rather uncomplicated strategies can be compared to a **conditional model 2** which implements the model of equation A3 without allowing for a variant by drug interaction. The full model of equation A3, including the variant by treatment interaction term, will henceforth be called **conditional model 3**. Together with the **prior to treatment** model (equation A2) an estimate of γ can be obtained use equation A1.

Standard error estimates

As described above and in the manuscript, an estimate of γ can be obtained as a simple sum of λ_t or as the sum of $\lambda_{t=T} + \sum_{t=0}^{T-1} \lambda_t \omega_{3t}$. When working with sample estimates, the standard errors of these sums can be estimated using, for example using bootstrap re-sampling (in the simulations we used 500 replication). Notice that the marginal model only has λ_T available. However because the *marginal model* does not condition on any previous $\boldsymbol{Y}_t$ measurements $\lambda_T = \gamma$ (in this case only), provided its described assumptions holds.

2 Data generating model

Simulated data were generated following the bottom right diagram of Figure 2 (main text). Specifically, phenotype observations $\boldsymbol{Y}$ were generated for $i = 1, \ldots, n$ subjects based on equations A2-A3 with error terms following a multivariate normal distribution $\mathcal{N}(\mathbf{0}, \boldsymbol{\Sigma}_y)$. Genetic exposures g_i were generated based on trinomial distribution with

probability q. The non-drug related environmental factors, represented by u_{it}, followed a multivariate distribution $\mathcal{N}(\boldsymbol{\mu}, \boldsymbol{\Sigma}_u)$. Treatment was generated as a series of Bernoulli trials with probability at $t = 0$

$$\text{logit}\,(p_{it=0}) = \alpha_0 + \alpha_1 y_{it=0} + \alpha_2 u_{it=0},$$

and for $(t > 0)$ additionally determined by preceding treatment

$$\text{logit}\,(p_{it}) = \alpha_0 + \alpha_1 y_{i,-t} + \alpha_2 u_{i,-t} + \alpha_3 d_{i,-t}.$$

3 Simulation scenarios

To evaluate the previously described GWAS modelling strategies we simulated data following the described data generating model. In scenarios 1 and 2 data were generated for $n = 5,000$ subjects included in a point-exposure study (i.e., where treatment was allocated once), with baseline data and follow-up data $t \in \{0, 1\}$. Specifically, in **scenario 1** an *RCT* was simulated with $\alpha_0 = \alpha_1 = \alpha_2 = \alpha_3 = 0$ resulting in a 1:1 treatment allocation scheme. Throughout the effect of $y_{i,-t}$ on y_{it} was set to $\omega_3 = 3$, the effect of u on y equalled $\omega_2 = -10$. Because scenario 1 pertains to a GWAS were treatment was randomly allocated there is no possibility of mediation, hence we focused on interaction iterating $\omega_4 \in \{0.5, 1.0, \ldots, 5\}$; with the genetic *(*direct) effect $\lambda = 0.5$, and treatment effect $\omega_1 = -10$.

In **scenario 2.A** a *nonrandomized* point-exposure study was simulated, letting d_i depend on $y_{it=0}$ and $u_{it=0}$ setting $\mathrm{e}^{\alpha_1} = 1.2, \mathrm{e}^{\alpha_2} = 1.5$, choosing a value of α_0 to ensure 1:1 treatment allocation. The following phenotype effects were used $\omega_1 = -10, \omega_2 = -10, \omega_3 = 3, \omega_4 = 0, \lambda = 0.5$, the impact of the direct genetic effect was evaluated by setting $\lambda \in \{0.0, 0.2, \ldots, 1.8\}$. Second, in **scenario 2.B** the impact of treatment was further explored by setting $\omega_1 \in \{0, -4, -8, \ldots, -36\}, \lambda = 0.5$, with the remaining parameters values as defined in scenario 2.A.

More general time-varying nonrandomized data were simulated in **scenario 3** with $t \in \{0, \ldots, 5\}$.Throughout scenario 3 the parameters values were chosen to be smaller than in scenarios 1 and 2, reflecting the presence of multiple time-points: $\omega_1 = -2, \omega_2 = -1, \omega_3 = 0.4, \mathrm{e}^{\alpha_1} = 1.05, \mathrm{e}^{\alpha_2} = 1.02, \mathrm{e}^{\alpha_3} = 1.2$. In **scenario 3.A** we assumed that all 6 measurement were observed, for example as might be possible with EHR. In **scenario 3.B** we assumed that only the baseline and last moment of follow-up were observed. Scenario 3.B is an example of (incorrectly) treating a longitudinal study as a point-exposure study with time invariant treatment.

In these scenarios g_i followed a trinomial distribution with $q = 0.30$, the diagonal of $\mathbf{\Sigma}_y$ equalled 10, its off-diagonal 0, and $\boldsymbol{\mu} = \mathbf{1}$. Diagonal values of $\mathbf{\Sigma}_\mu$ equalled 1, and its off-diagonal 0.70 (resulting in a correlation of 0.70). While $\mathbf{\Sigma}_y$ implies *conditional* independence between y_t, the first order Markov relation of equation A3 results in marginal correlations between y_t. Throughout ω_{0t} was chosen to ensure the marginal mean of y would be constant across t.

4 Appendix Tables and Figures

Appendix Table A: variants used in a genetic analysis of HbA_{1c}

**rsID**	**Minor Allele**	**Minor Allele Frequency**
rs10203174	T	0.13
rs10213440	C	0.19
rs10269209	T	0.32
rs10278336	G	0.43
rs10401969	C	0.10
rs10811661	C	0.20
rs10830963	G	0.26
rs10842994	T	0.19
rs10965250	A	0.19
rs11063069	G	0.17
rs1111875	T	0.44
rs11212617	C	0.44
rs11257655	T	0.23
rs11634397	A	0.33
rs11651755	C	0.46
rs11703495	T	0.09
rs11708067	G	0.25
rs11717195	C	0.25
rs11873305	C	0.06
rs11899863	T	0.09
rs11920090	A	0.14
rs121918407	C	0.50
rs12208357	T	0.06
rs12242953	A	0.43
rs12427353	C	0.18
rs12497268	C	0.16
rs12571751	G	0.47
rs12899811	G	0.32

Appendix Table A: variants used in a genetic analysis of HbA_{1c} *(continued)*

**rsID**	**Minor Allele**	**Minor Allele Frequency**
rs12943590	A	0.24
rs12970134	A	0.25
rs13233731	A	0.44
rs13292136	T	0.08
rs13389219	T	0.39
rs1387153	T	0.27
rs1470579	C	0.34
rs1496653	G	0.20
rs1531343	C	0.10
rs1535500	T	0.49
rs1552224	C	0.16
rs163184	T	0.50
rs16861329	T	0.14
rs16927668	T	0.22
rs17168486	T	0.18
rs17584499	T	0.23
rs17791513	G	0.09
rs1801214	C	0.38
rs1801282	G	0.09
rs1802295	T	0.31
rs2007084	A	0.08
rs2028299	C	0.32
rs2075423	T	0.34
rs2191349	G	0.43
rs2261181	T	0.09
rs2289669	A	0.41
rs231361	A	0.28
rs231362	G	0.50
rs243019	C	0.32
rs243021	A	0.49

Appendix Table A: variants used in a genetic analysis of HbA_{1c} *(continued)*

**rsID**	**Minor Allele**	**Minor Allele Frequency**
rs243088	T	0.50
rs2447090	G	0.32
rs2796441	A	0.36
rs2943640	A	0.33
rs316019	A	0.12
rs340874	T	0.08
rs391300	T	0.39
rs3923113	C	0.38
rs4253762	G	0.09
rs4299828	G	0.21
rs4402960	T	0.34
rs4410242	A	0.26
rs4430796	G	0.46
rs4457053	G	0.30
rs4502156	C	0.42
rs459193	A	0.23
rs4607517	A	0.18
rs4720572	C	0.36
rs4724512	G	0.25
rs4760790	A	0.26
rs4760915	T	0.27
rs4810083	T	0.44
rs4812829	A	0.21
rs5015480	T	0.44
rs5215	C	0.38
rs6008976	A	0.49
rs6467136	A	0.45
rs6519979	C	0.50
rs662301	T	0.08
rs6769511	C	0.35

Appendix Table A: variants used in a genetic analysis of HbA_{1c} *(continued)*

**rsID**	**Minor Allele**	**Minor Allele Frequency**
rs6795735	T	0.41
rs6819243	C	0.49
rs683369	G	0.25
rs6878122	G	0.30
rs6958502	A	0.17
rs6959643	T	0.17
rs6960043	T	0.48
rs6963810	G	0.42
rs7041847	G	0.48
rs7163757	T	0.41
rs7177055	G	0.29
rs7178572	A	0.28
rs73886756	A	0.50
rs7569522	A	0.40
rs7578326	G	0.33
rs7593730	T	0.25
rs7612463	A	0.11
rs7756992	G	0.33
rs7845219	C	0.44
rs784888	C	0.49
rs7903146	T	0.35
rs7955901	C	0.48
rs7957197	A	0.17
rs8042680	A	0.33
rs8065082	T	0.43
rs8108269	G	0.31
rs8182584	T	0.40
rs831571	T	0.17
rs849134	G	0.43
rs849135	A	0.42

Appendix Table A: variants used in a genetic analysis of HbA_{1c} *(continued)*

**rsID**	**Minor Allele**	**Minor Allele Frequency**
rs9368222	A	0.32
rs9470794	C	0.08
rs972283	A	0.44
rs9936385	C	0.43

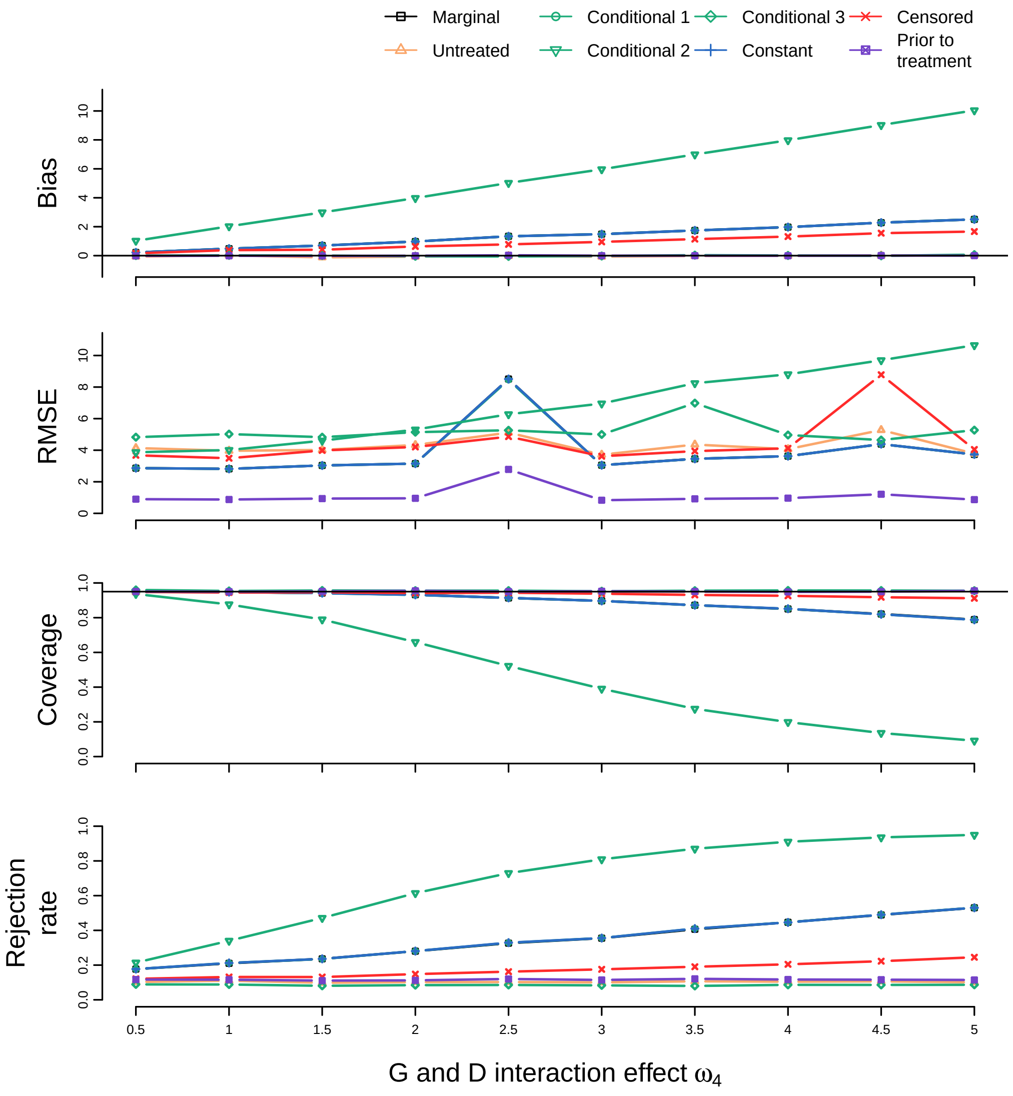

Appendix Figure A: Simulation results for scenario 1 where the life-time variant-to-phenotype association was modified by treatment (a variant by treatment interaction); residuals were sampled from a t-distribution with 2 degrees of freedom. Nb. Simulations were repeated 10,000 times. See Table 1 for a description of the modelling strategies used.

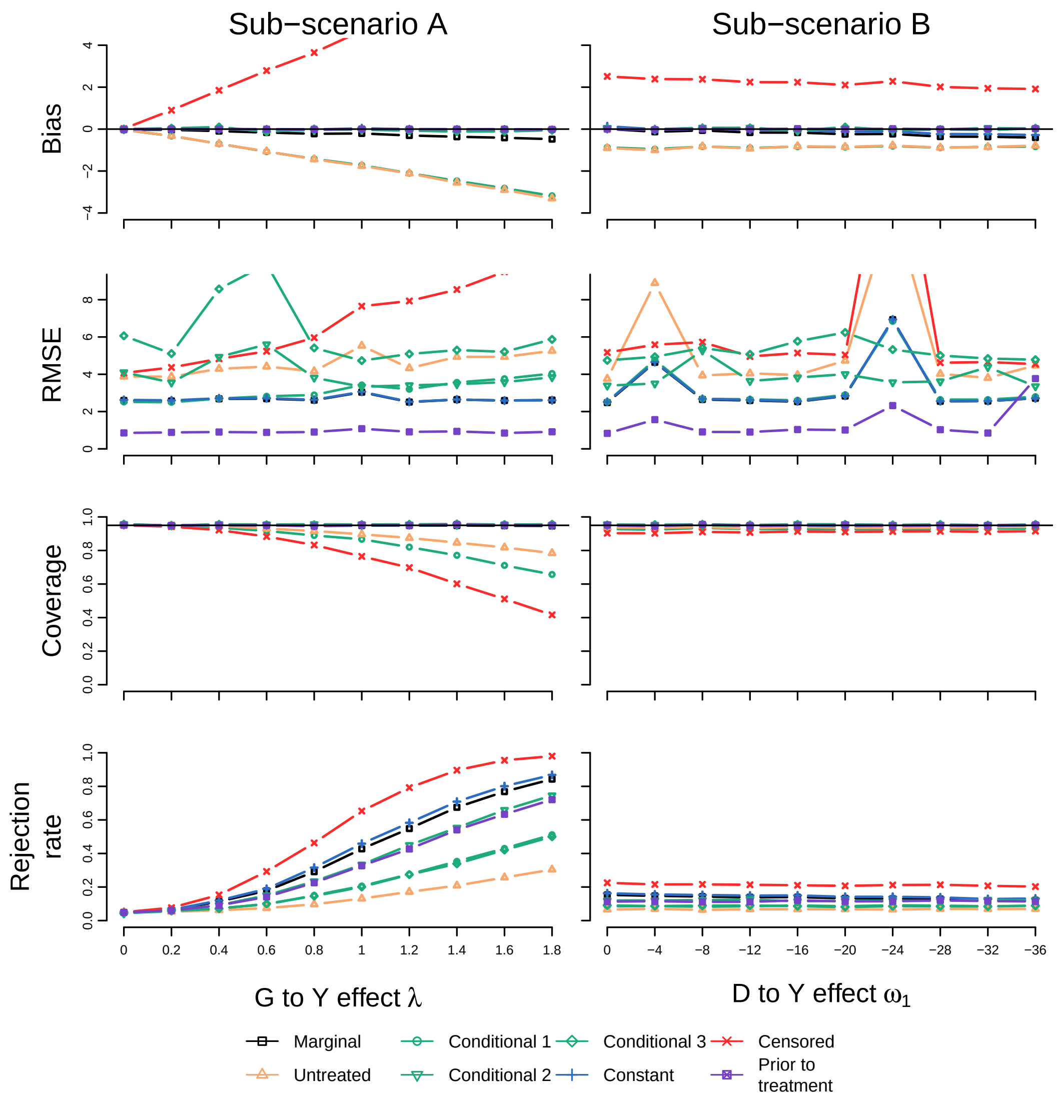

Appendix Figure B: Simulation results for scenario 2 where the life-time variant-to-phenotype association was mediated by treatment; residuals were sampled from a t-distribution with 2 degrees of freedom. Nb. Simulations were repeated 10,000 times. See Table 1 for a description of the modelling strategies used.

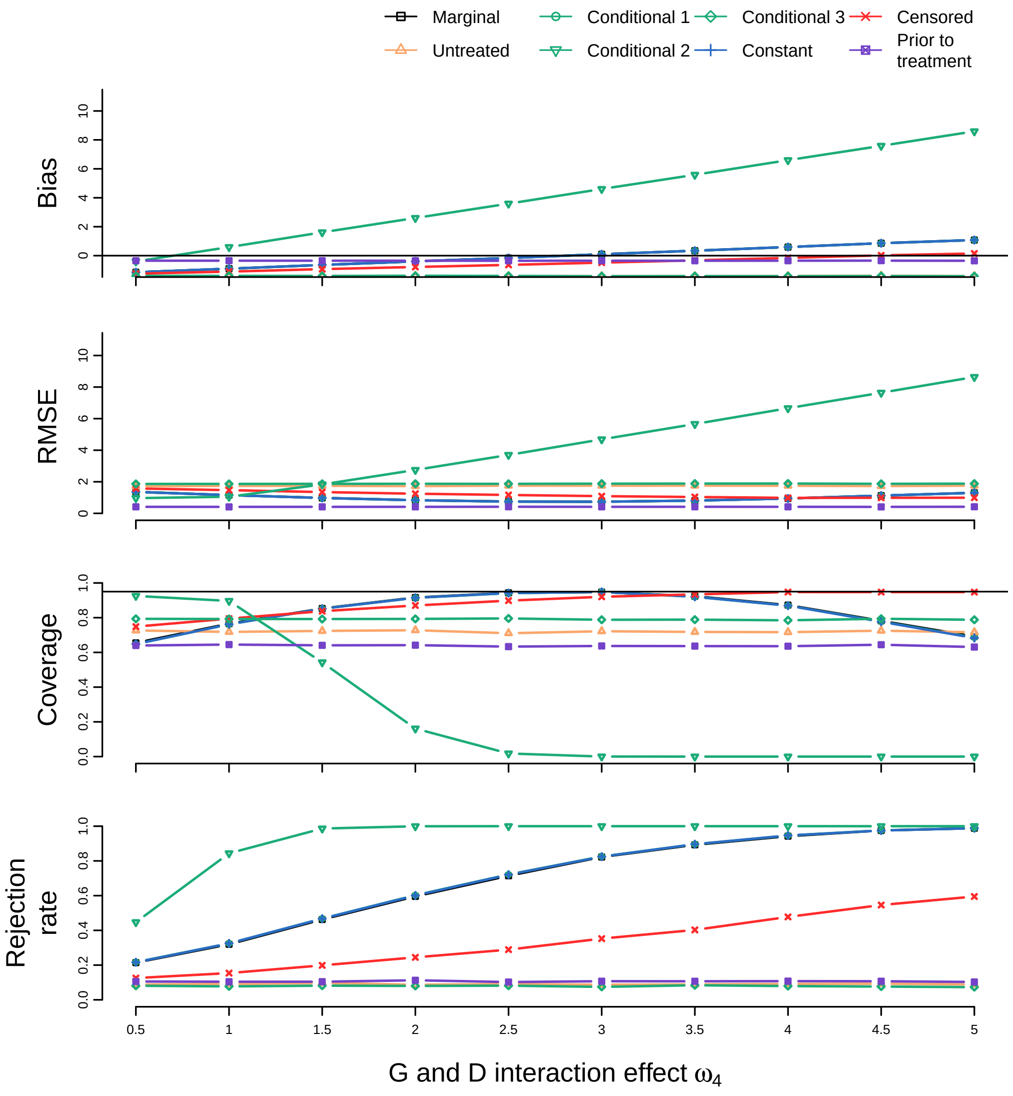

Appendix Figure C: Simulation results for scenario 1 where the life-time variant-to-phenotype association was modified by treatment (a variant by treatment interaction); outcome data were simulated under a dominant genetic model. Nb. Simulations were repeated 10,000 times. See Table 1 for a description of the modelling strategies used.

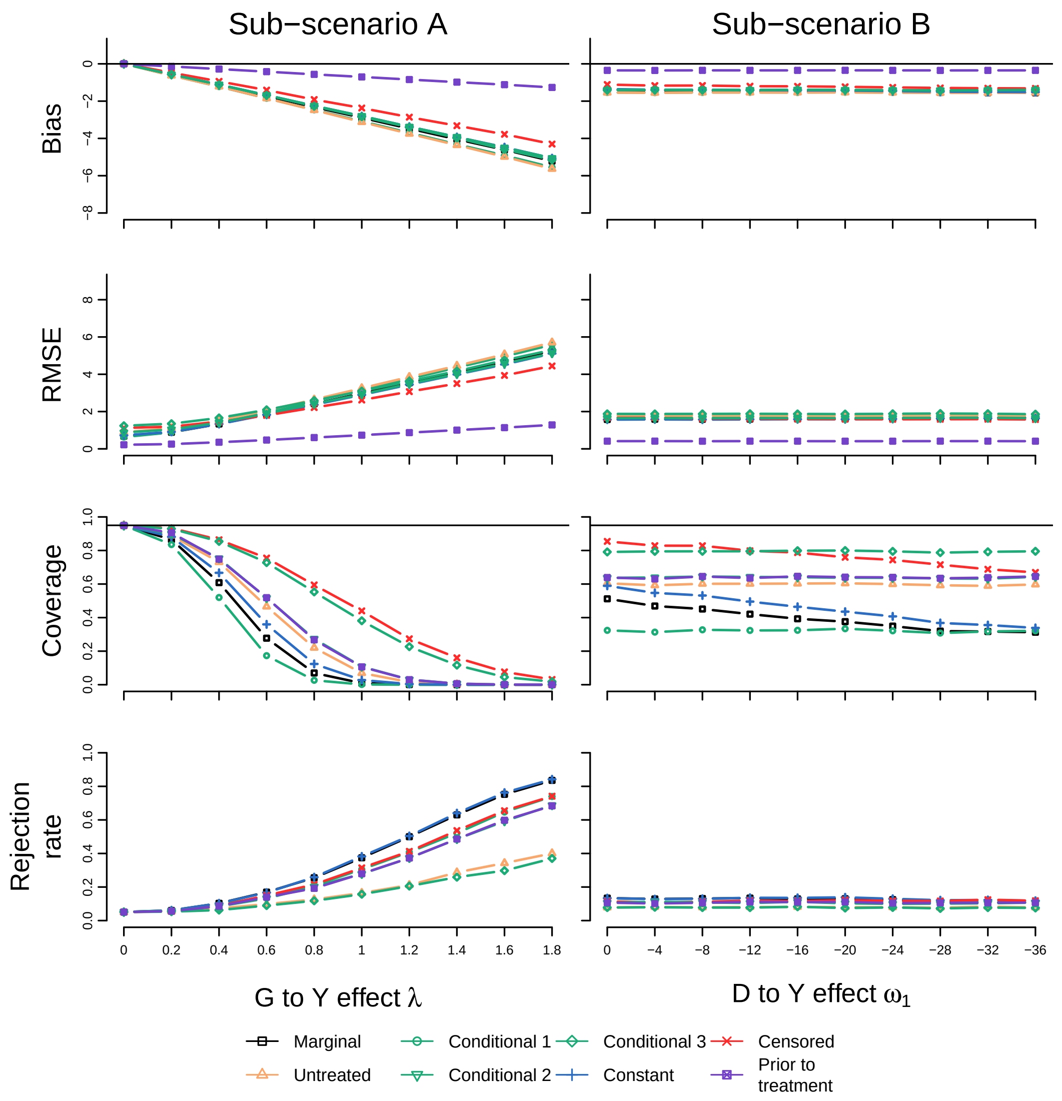

Appendix Figure D: Simulation results for scenario 2 where the life-time variant-to-phenotype association was mediated by treatment; outcome data were simulated under a dominant genetic model. Nb. Simulations were repeated 10,000 times. See Table 1 for a description of the modelling strategies used.

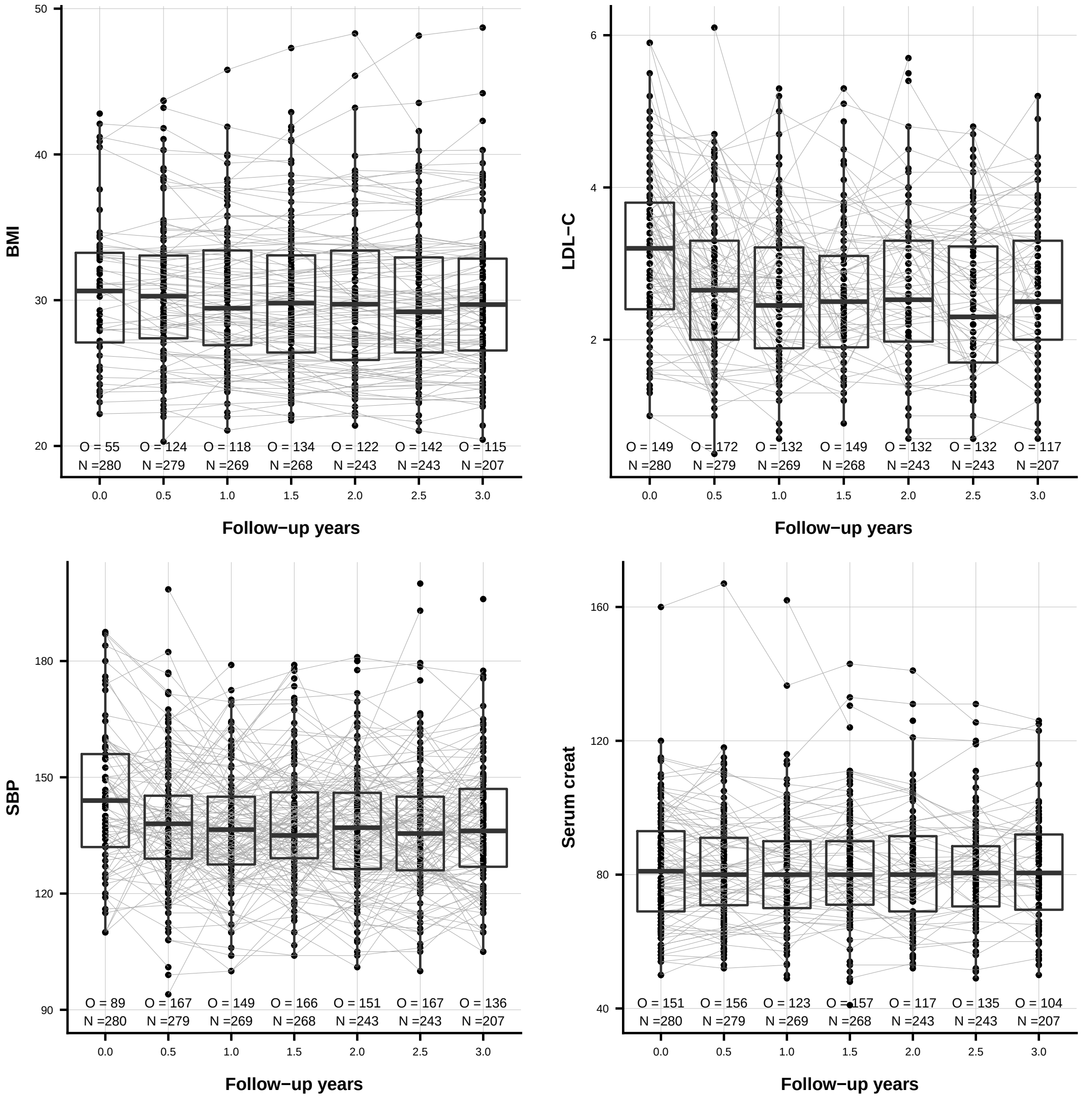

Appendix Figure E: longitudinal development of T2DM relevant biomarkers. Nb. grey lines indicate individual trajectories; O and N, observed, and total number of subjects available.

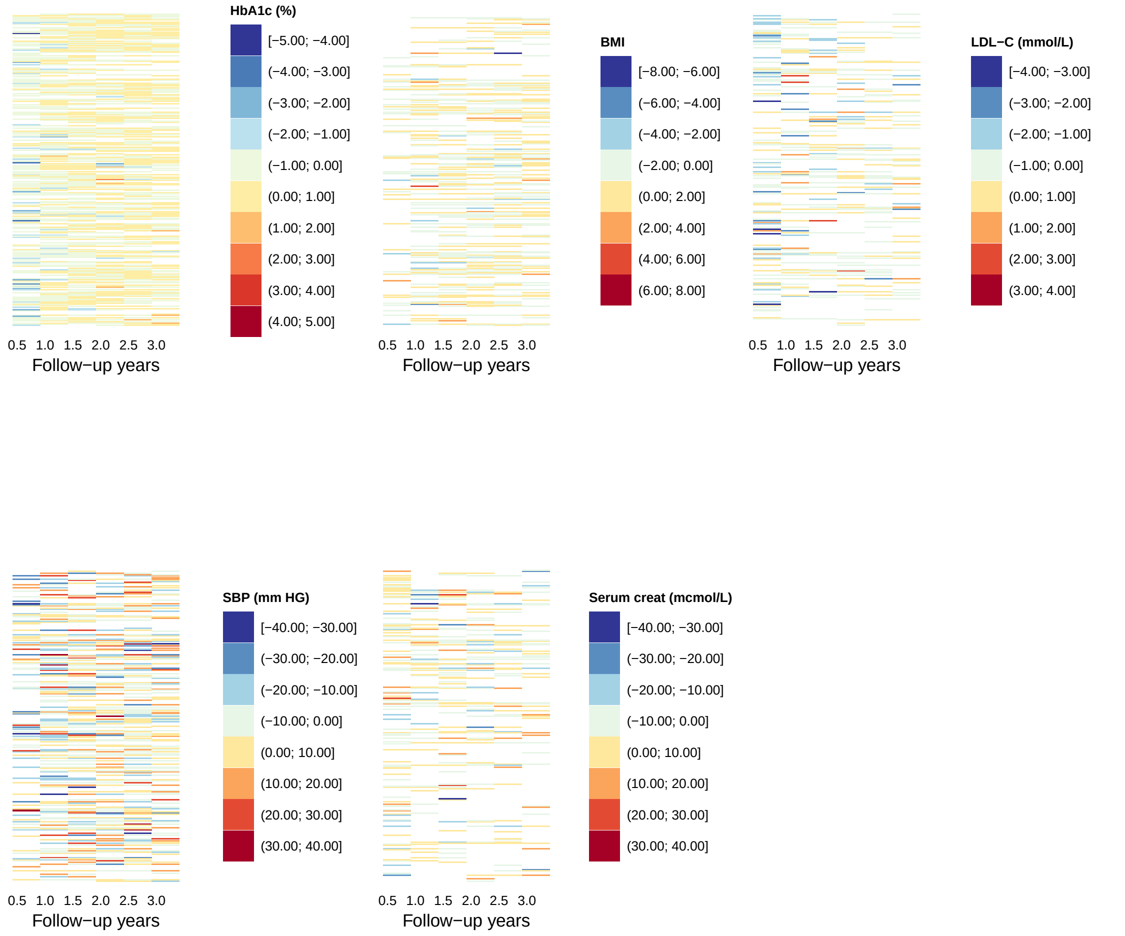

Appendix Figure F: heatmap of longitudinal changes for T2DM relevant biomarkers; patients are indexed on the rows, values (colours) represent the difference between the current and previous (in the preceding 6 months) measurement.

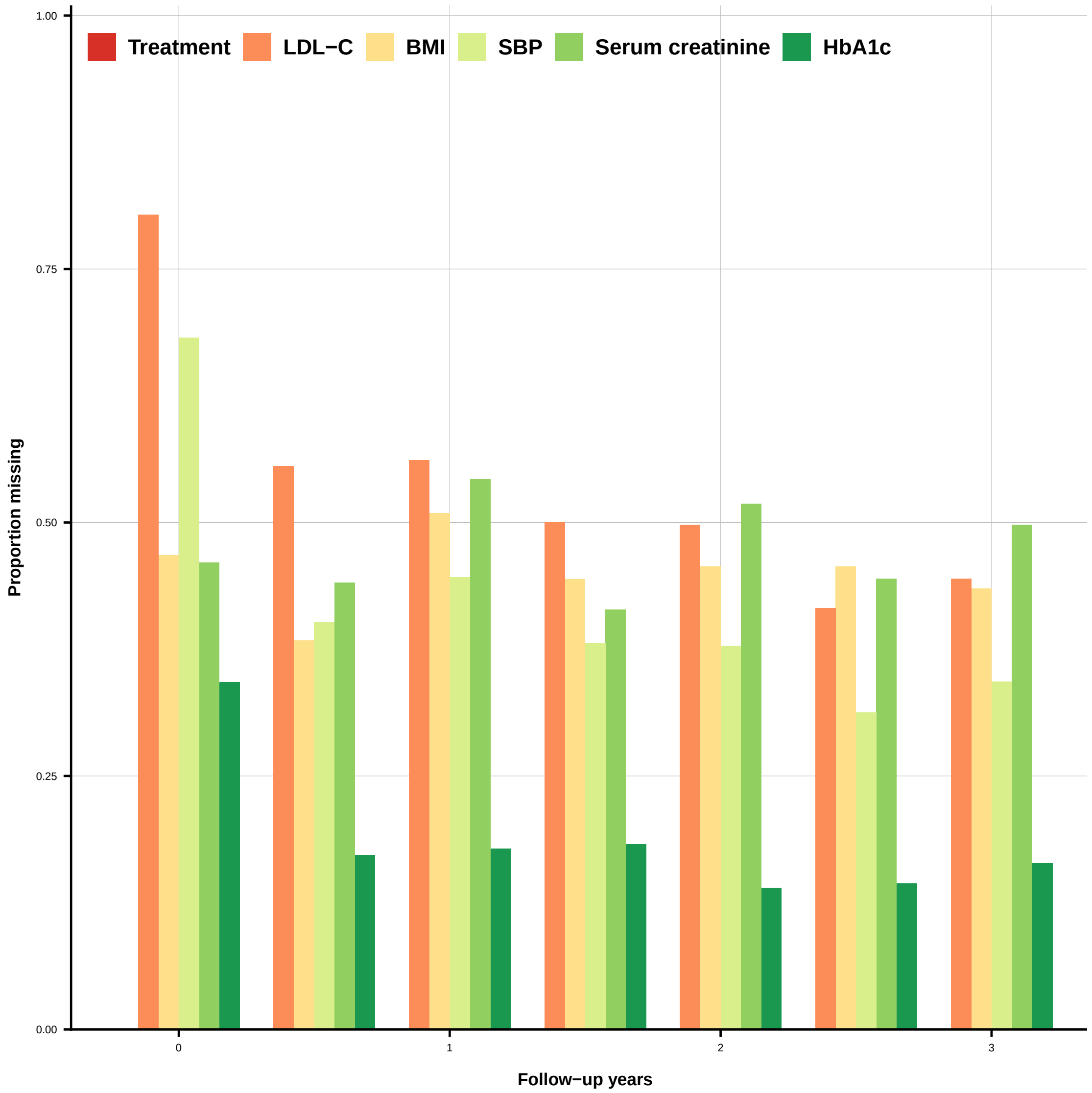

Appendix Figure G: proportion missing observations across follow-up time (notice that treatment did not have any missing observations).

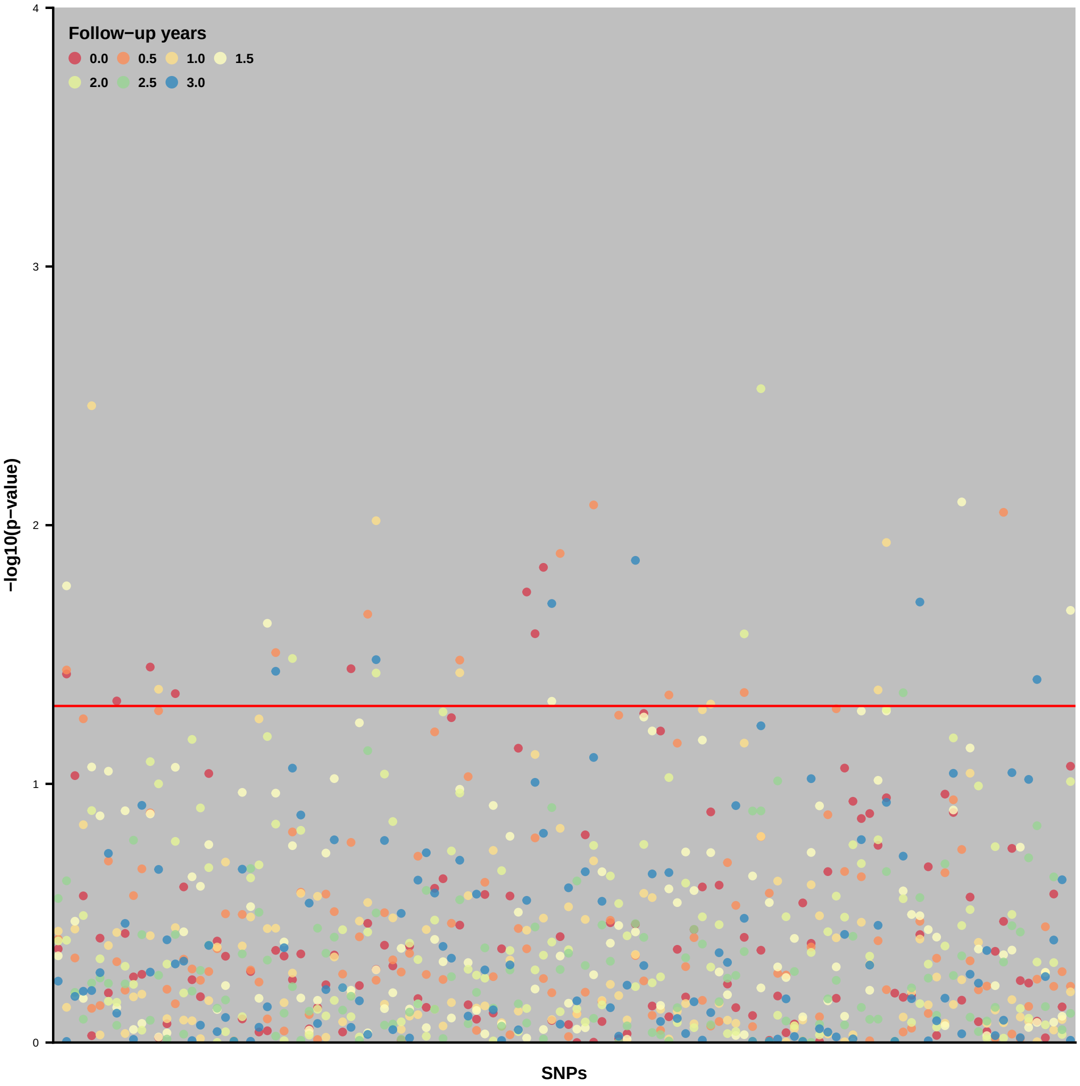

Appendix Figure H: a Manhattan plot of the SNP associations with missing HbA_{1c} values across follow-up time. Nb. The horizontal red line indicates a $-log_{10}$ p-value of 0.05

Appendix Table B: p-values for the relation with a HbA_{1c} complete case indicator across 6-months follow-up periods and baseline (0)

**Period**	**0**	**1**	**2**	**3**	**4**	**5**	**6**
**BMI**	0.47	0.32	0.07	0.09	0.07	0.11	0.97
**LDL-C**	0.48	0.62	0.39	0.50	0.45	0.42	0.33
**SBP**	0.44	0.09	0.94	0.63	0.09	1.00	0.04
**Creatinine**	0.03	0.79	0.93	0.97	0.91	0.48	0.19
**Treatment**	< 0.01	< 0.01	< 0.01	0.11	0.03	0.14	0.99

Appendix Table C: The average mean difference, and percentage number of significant associations (using two thresholds) of a 122 variant association with longitudinal HbA_{1c} stratified by 8 different treatment modelling strategies.

	**Average mean difference (sd)**	**Percentage p-value < 0.05**	**Percentage p-value $< 8 \times 10^{-8}$**
Marginal	-0.02 (0.62)	27.87	4.92
Untreated	0.12 (1.20)	29.90	2.06
Conditional 1	-0.01 (0.64)	24.59	4.10
Conditional 2	0.00 (0.25)	9.84	2.46
Conditional 3	0.05 (1.05)	34.43	3.28
Constant	-0.02 (0.65)	22.95	4.10
Censored	-0.01 (0.62)	25.41	3.28
Prior Treatment	0.01 (0.16)	4.10	0.00

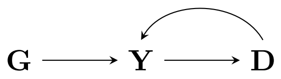

Appendix Figure I: a directed cyclic graph depicting Tobin et. al. [1] data generation model for a study with a genetic ($\boldsymbol{G}$) exposure, treatment ($\boldsymbol{D}$) and an outcome phenotype ($\boldsymbol{Y}$).

References

[1] Martin D Tobin, Nuala A Sheehan, Katrina J Scurrah, and Paul R Burton. “Adjusting for treatment effects in studies of quantitative traits: antihypertensive therapy and systolic blood pressure”. In: *Statistics in medicine* 24.19 (2005), pp. 2911–2935.
